# Invasion origin, rapid population expansion, and the lack of genetic structure of cotton bollworm (*Helicoverpa armigera*) in the Americas

**DOI:** 10.1002/ece3.5123

**Published:** 2019-06-17

**Authors:** Rogério Martins Gonçalves, Thiago Mastrangelo, José Carlos Verle Rodrigues, Daniel Fernando Paulo, Celso Omoto, Alberto Soares Corrêa, Ana Maria Lima de Azeredo‐Espin

**Affiliations:** ^1^ Department of Genetics, Evolution and Bioagents, Institute of Biology State University of Campinas (UNICAMP) Campinas Brazil; ^2^ Graduate Program in Genetics and Molecular Biology, Institute of Biology State University of Campinas (UNICAMP) Campinas Brazil; ^3^ Centre for Nuclear Energy in Agriculture University of São Paulo (USP) Piracicaba Brazil; ^4^ Center for Excellence in Quarantine & Invasive Species University of Puerto Rico (UPR) San Juan Puerto Rico; ^5^ Department of Entomology and Acarology Luiz de Queiroz College of Agriculture, University of São Paulo (USP/ESALQ) Piracicaba Brazil

**Keywords:** Americas, cotton bollworm, invasion route, invasive pest, putative hybrids

## Abstract

In 2013, *Helicoverpa armigera* (Hübner) (Lepidoptera: Noctuidae) was officially declared as present in Brazil and, after two years, the species was detected in the Caribbean and North America. Information on genetic features and accurate distribution of pests is the basis for agricultural protection policies. Furthermore, such knowledge is imperative to develop control strategies, understand the geographical range, and genetic patterns of this species in the Americas. Here, we carried out the widest sampling of *H. armigera* in the South American continent and Puerto Rico, after we estimated the diversity, demographic parameters, and genetic structure. The Internal Transcribed Spacer 1 (ITS1) nuclear marker was used to investigate the presence of putative hybrids between *H. armigera* and *H. zea,* and they were observed at a frequency of 1.5%. An ABC analysis, based in COI gene fragment, suggested Europe as the origin of South America specimens of *H. armigera*and following a movement northward through the Caribbean. Three mtDNA genes and three nDNA markers revealed high genetic diversity distributed without the defined population structure of *H. armigera* in South America. Most of the genetic variation is within populations with a multidirectional expansion of *H. armigera* among morphoclimatic regions. High genetic diversity, rapid population expansion, and hybridization have implications for pest management since they suggest that adaptive alleles are spread through wide areas in South America that favor rapid local adaptation of *H. armigera* to new and disturbed environments (e.g., in agricultural areas).

## 1 INTRODUCTION

The agribusiness sector accounts for more than 20% of the Brazilian gross domestic product (GDP) (IBGE, 2016). Nevertheless, this sector suffers economic losses of US$ 17.7 billion per year due to pest damage on 35 major crops (Oliveira, Auad, Mendes, & Frizzas, 2014). Plant protection policies to control insect pests are limited by lack of efficient management strategies and new population outbreaks caused by ecologic disturbances and invasive pests. The introduction of exotic pests has been an enduring problem in the world, causing large negative impacts over the past three decades (Lopes‐da‐Silva, Sanches, Stancioli, Alves, & Sugayama, 2014). This is the case of the phytosanitary crisis caused by the Old World cotton bollworm, *Helicoverpa armigera* (Hübner) (Lepidoptera: Noctuidae), in Brazil and its dispersion northward to the Americas.

**Figure 1:**
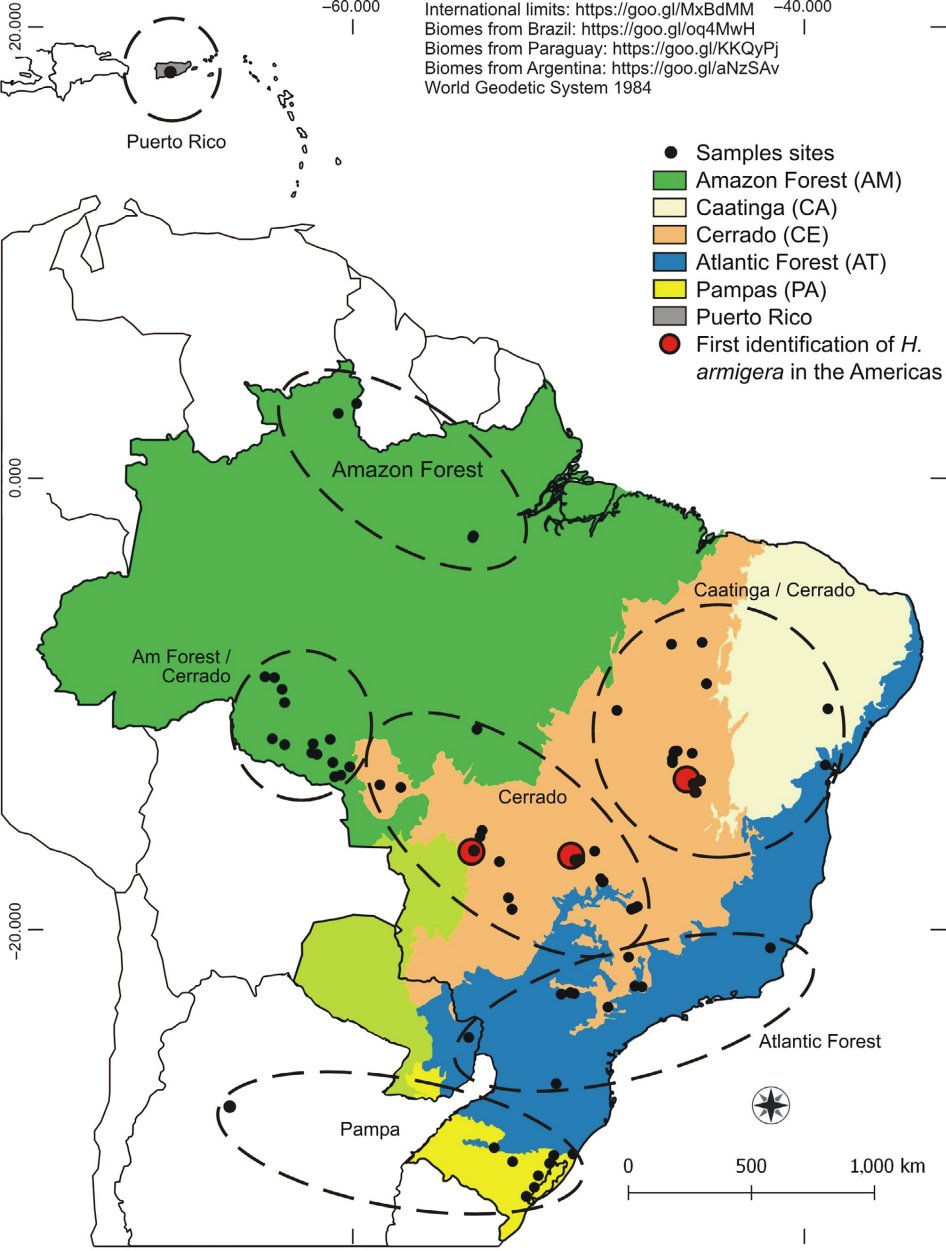
Collection points of *Helicoverpa armigera* sampled in South America and Puerto Rico showing the morphoclimatic regions where the samples were obtained. Dashed lines indicate the sites grouping used in the analyses. Red dots indicate the three farms where *H. armigera* was identified for the first time on the American continent by Czepak et al. (2013)

After the first record of *H. armigera* in Brazil in January 2013 in Goiás, Mato Grosso, and Bahia States (red dots in Figure 1; Czepak, Albernaz, Vivan, Guimarães, & Carvalhais, 2013; Tay et al., 2013), *H. armigera* population outbreaks occurred in the same year in a wide geographical area, especially the row crop areas of the northeastern and central regions of the country and constantly associated with reports of control failures of pyrethroid pesticides (Durigan et al., 2017; EMBRAPA, 2013). Strategic control tactics to contain or eliminate invasive pests depend on an accurate spatial characterization of the invasion and dispersion processes of the species in its new territory. However, *H. armigera* is morphologically similar to the native pest *H. zea* (Boddie) (Lepidoptera: Noctuidae). In larval stages, they are morphologically indistinguishable, which made data collection concerning geographical distribution and dispersion of this pest in Brazil difficult (Pogue, 2004). Furthermore, hybridization events between *H. armigera* and *H. zea* are a real scenario under natural conditions (Anderson et al., 2018; Laster & Hardee, 1995; Laster & Sheng, 1995; Leite, Corrêa, et al., 2017).

Despite the absence of an official record about specific invasion routes of *H. armigera* to Brazil, sequential investigations rapidly started, indicate new reports of *H. armigera* in the Americas. Initially, outbreaks began to be reported in other South American countries (Arnemann et al., 2016; Murúa et al., 2014), then to Caribbean (NAPPO, 2014) and, finally, Florida, USA, in 2015 (APHIS, 2015; Hayden & Brambila, 2015). Around three years after being officially reported, *H. armigera* had almost reached the entire American continent. Its rapid geographical expansion is favored by high dispersion capacity mediated by the wind, mild winter temperatures, and the intense cropping systems, which provide a wide availability of food and high reproductive rates during the four seasons of the year. All detections of *H. armigera* in Puerto Rico have been reported in the southern drier region of the island that concentrates on year‐round production of vegetable (i.e., tomato, peppers) and is one of the largest winter nursery breeding operations in the United States for major row crops (i.e., soybean, corn, cotton, and sorghum) (Gerrero, 2009).

Recent population studies revealed a high genetic diversity of *H. armigera* populations in Brazil and genetic similarity among Brazilian individuals with populations originating in Europe, Africa, and Asia (Anderson, Tay, McGaughran, Gordon, & Walsh, 2016; Leite, Alves‐Pereira, Corrêa, Zucchi, & Omoto, 2014; Mastrangelo et al., 2014; Tay et al., 2017). The high genetic diversity of *H. armigera* increases doubts surrounding an invasion process and population dynamics of *H. armigera* in Brazil, since high genetic diversity is not expected in invasive insects due to the founder effect of invasion processes.

Hybridization is an important event in the process of colonization of new ecosystems by invasive species because they may receive new and favorable alleles of a native species (Lewontin & Birch, 1966; Mesgaran et al., 2016). Fertile hybrids between *H. armigera* and *H. zea*, in laboratory conditions, were initially reported by Laster and Hardee (1995) and Laster and Sheng (1995). However, due to the reproductive isolation resulting from the large distance of occurrence between these two species, hybrids had never been observed under natural conditions. This recurrent question has now arisen after the *H. armigera* invasion in the Americas and the occurrence of these two species in the same geographical region with a huge impact on pest management.

Here, we present the largest temporal and geographical sampling of *H. armigera* made in the Americas since its invasion, providing an important dataset that allows new insights into little‐understood issues like geographical distribution, dispersive traits, and genetic source of this invasion in the American territory. Furthermore, we used different population genetic approach methods (ABC analysis, nDNA, mitochondrial, and ITS markers) still not used to reveal the demographic events that shaped the *H. armigera* populations in South America, which are still suffering from significant data scarcity at the broader geographical level. Thus, we used seven different nuclear and mitochondrial molecular markers in order to (a) elucidate the invasion routes of *H. armigera* populations in South America, (b) assess the demographic parameters and population structure of *H. armigera* populations from Brazil, its bordering countries, and Puerto Rico, (c) assess the gene flow among *H. armigera* population collected in different Brazilian regions, and finally, (d) investigate the presence of putative hybrids between *H. armigera* and *H. zea* in Brazil. Such information is important in helping to define immediate control strategies and long‐term solutions for *Helicoverpa* management in South America and providing substantial information to support prevention and preparedness for the imminent arrival of the pest in the main North America agricultural regions.

## 2 MATERIAL AND METHODS

### 2.1 Samples of *Helicoverpa armigera* and DNA extraction

*Helicoverpa*spp. individuals were collected at 69 geographical sites from Brazil, Paraguay, Argentina, and Puerto Rico (Caribbean) during the 2013–2016 harvests (Figure 1; a detailed list of collection sites is available on Dryad repository: https://doi.org/10.5061/dryad.rd1570s). Larvae collected directly from host plants and moths from delta sticky and bucket traps baited with pheromone lures were immediately preserved at −20°C in 100% ethanol until DNA extraction. Total DNA was extracted from the abdomen of the specimens using the standard phenol:chloroform method, adapted for microcentrifuge tubes (Lyra, Klaczko, & Azeredo‐Espin, 2009). The pellet was resuspended in 100 µl of TE buffer and stored at −20ºC.

### 2.2 Mitochondrial markers

We used three partial sequences of mitochondrial genes: cytochrome *c* oxidase subunit I (COI; *COI*F/*COI*R; Li et al., 2011), cytochrome *c* oxidase subunit II (COII; A‐tLEU/B‐tLYS; Liu & Beckenbach, 1992), and cytochrome *b* (Cyt *b*; Harm CB‐J‐10769/Harm CB‐N‐11325; primers designed in this study) (Appendix A). The PCR were conducted in a final volume of 25 μl for mtDNA with 25 ng of total DNA, 1.5 U of TaqDNA polymerase (Fermentas International Inc., Canada), 56 µM of dNTPs, 2.5 mM of MgCl_2_, 0.3 mg/ml of0020BSA, 10× TaqBuffer, and 0.16 µM of each primer. Successful amplifications were purified with the Illustra^TM^GFX^TM^ kit (GE Healthcare, Bucks, UK), cycle sequencing reactions were completed using the BigDye^®^ Terminator v3.1 kit (Thermo Fisher Scientific Inc., Waltham, EUA), and bidirectional sequenced by the ABI 3730xl DNA Analyzer sequencer (Applied Biosystems, Foster City, EUA).

Geneious 6.0.6 software package (Biomatters Ltd, Auckland, NZ) was used to assemble the nucleotide sequences into a contig for each specimen. The generated sequences were aligned employing the multiple sequences alignment algorithm implemented in Clustal Omega (Sievers et al., 2011), manually edited and the translated sequences were checked for the presence of premature stop codon in MEGA 5.1 (Tamura et al., 2011). Sequences were independently identified by BLAST 2.3.0 search (www.ncbi.nlm.nih.gov/BLAST/) against sequences stored in NCBI GenBank database (http://www.ncbi.nlm.nih.gov/genbank/). In this step, COI also were used to *H. armigera* species identification.

#### 2.2.1 Inferring Brazilian *H. armigera* origin

In short, we used an Approximate Bayesian Computation (ABC) approach (Csilléry, Blum, Gaggiotti, & François, 2010), designed here for the first time for *H. armigera*, to investigate the origin of its invasion in South America. We designed demographic and coalescent models (see below) that were used to generate a simulated data distribution on which summary statistics were calculated. A rejection algorithm retained the statistics, among all the models, that have the smallest Euclidean distance to the empirical data, obtained from the COI dataset. Then, the model that best recovered the empirical data presented the highest Posterior Probability value (PP).

We used 36 COI mtDNA sequences from the Americas (the first two sequences obtained in each Brazilian state and in each South American country), 40 from Asia (28 from Li et al., 2011; one from Cho et al., 2008: EU768935, and unpublished: HM854928–HM854932, FN908003, FN908011, FN908013, FN908016, JX532104, AB620128), 11 from Europe (unpublished: GU686757, KJ460247, FN908014, FN908015, FN907996–FN908002), and five from Africa (unpublished: FN907995, FN908005, FN908006, FN908017, FN908018). We chose not to use all sequences obtained in our work in order to have a similar number of sequences among the continents.

**Figure 2:**
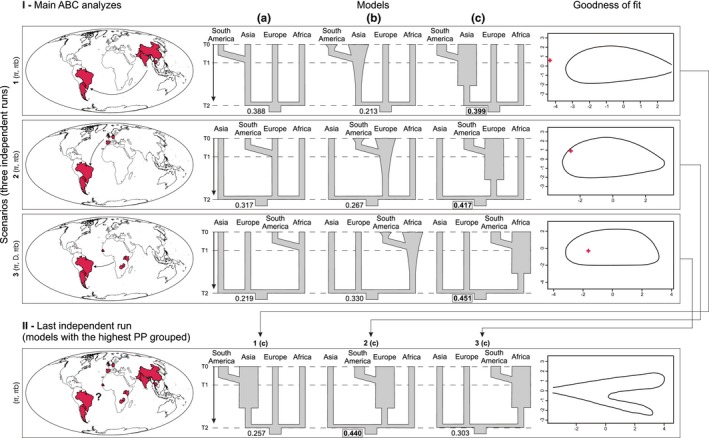
An ABC analysis performed to investigate founder effect in *H. armigera* samples from South America. I—In horizontal, three independent ABC runs of scenarios based in COI marker represent individuals coming from (1) Asia; (2) Europe, and (3) Africa. Arrows represent time into past, T0 = present, T1 = divergence time, and T2 = coalescent time. Summary statistics vector most informative for the data are between parentheses. In vertical and inside each scenario, three models illustrate (a) constant population size; (b) exponential population growth, and (c) very rapid population expansion. Numbers at the bottom of the graph correspond to posterior probabilities (PP) while bold numbers inside the rectangle represent the highest PP found in each scenario. The goodness of fit test of the scenarios is shown on the right; Red cross: observed SuSt. II—Last independent ABC run for the scenarios comparison containing the best models obtained before (1c, 2c, 3c)

Initially, three simple and specific scenarios were designed (independent ABC runs) testing three similar models in each as shown in Figure 2‐I. Each of the three scenarios simulates different hypotheses for the founder effect with individuals going to the American continent from (1) Asia, (2) Europe, and (3) Africa. The three models in each scenario assume very recent divergence between sequences from specimens collected in South America versus samples from one of the other three continents. The models differ in past demographic events, adding (a) a constant population size, (b) exponential population growth, and (c) very rapid population expansion before and after the divergence. We did not incorporate migration into our models even though we tested populations scattered on distant continents, but it is a question that could be explored in the future. The ABC approach is a robust method of analysis of situations with limited gene flow (Camargo, Morando, Avila, & Sites, 2012).

The ABC analyses were performed separately for each scenario in the *ms* 20161016 coalescent sampler (Hudson, 2002) to construct a prior distribution of simulated datasets. For each scenario, a custom Python™ script was designed with 300,000 data simulations. All scripts used in the ABC analysis and mentioned below are deposited in the Dryad repository: https://doi.org/10.5061/dryad.rd1570s. For simulations of the models, the values of the parameters were sampled randomly within the intervals whose maximum limits were calculated as follows: Wang, Fan, Owada, Wang, and Nylin (2014) detected the rate of 0.0115 substitutions/site/million years for COI in Noctuidae, thus we considered 0.75 million years (divergence with *H. zea* was 1.5 million years ago; Mallet, Korman, Heckel, & King, 1993) as the maximum coalescence time of the sequences, five generations per year (Ge, Chen, Parajulee, & Yardim, 2005; Naseri, Fathipour, Moharramipour, & Hosseininaveh, 2011) and theta‐W = 4.123, which was obtained in DnaSp v5 (Librado & Rozas, 2009) from the 626bp COI dataset long with 92 sequences. The formula Theta = *Neµ* was calculated, *that is*, 4.123 = Ne (0.0115/10^6^. 626 pb); 4.123 = Ne(7.199/10^6^); Ne = 572,718.43, and tau was found as number of generations/2Ne; 3,750,000/1.145.436,86 = 3.274. The following observed Summary Statistics (SuSt) were calculated in DnaSp v5: nucleotide diversity (*π*), segregating sites (SS), Tajima's *D* (*D*), nucleotide diversity within each population (*πw*), and nucleotide diversity between each pair of populations (*πb*). For the simulated datasets, the same SuSt were calculated with the software Sample Stats (Hudson, 2002) and msSS.pl (a Perl script written by N. Takebayashi, available at: http://raven.iab.alaska.edu//ntakebay/teaching/programming/coalsim/scripts/msSS.pl).

After obtaining the prior distribution, the goodness of fit test was performed in the R package ABC (Csilléry, François, & Blum, 2012) with the *gfitpca* function to evaluate how well the simulated models fit the observed data. A good fit occurs when the observed SuSt is positioned within the simulated SuSt. Next, all possible combinations of two or more simulated SuSt were grouped into vectors. A rejection step was conducted with a Python script in the Euclidean distance C program msReject (http://msbayes.sourceforge.net/) using 10 simulations from the prior distribution of each model treated as empirical data to find the vector that best detects the true model (Tsai & Carstens, 2013). Then, these vectors were employed to find the 2.5% and 97.5% weighted values of each parameter in the R package ABC which were, respectively, the minimum and maximum new limits of these parameters in the main ABC analysis.

Therefore, the main ABC analysis was performed as described above, but now with 900,000 data simulations and restricted parameters. The model most supported by the data in each scenario was selected using msReject to retain the posterior distribution of 0.001 of the simulations closest to summary statistics from the empirical data. Again, only the vector selected previously was used. Lastly, the models with the highest PP values found in each of the three scenarios were carried out in a final independent run (Figure 2‐II), applying a hierarchical procedure similar to Fagundes et al. (2007), to infer the most likely model among the scenarios. This new ABC analysis and the rejection step were performed as described above, but with 3,000,000 data simulations and restricted parameters.

#### 2.2.2 Genetic diversity, demography, and population structure in Brazil

A total of 303 *H. armigera* individuals were used in this population study. Mastrangelo et al. (2014) previously sequenced 65 individuals for COI and COII mtDNA genes and, here, we added Cyt *b* gene to these individuals. The other 238 *H. armigera* individuals were sequenced for three mtDNA genes: COI, COII, and Cyt *b*. We concatenated the three genes, COI (658 pb), COII (554 pb), and Cyt *b* (434 pb) in a 1,646 bp‐long sequence for each of the 303 individuals, generating a total of 54 mtDNA haplotypes. The genetic diversity, demography, and population structure indices were estimated according to the morphoclimatic regions of the individuals (Ab'Saber, 1967; IBGE, 2016): 1—Amazon Forest biome (AM); 2—Amazon Forest transition with Cerrado biome (AM/CE); 3—Cerrado biome (CE); 4—Cerrado transition with Caatinga biome (CE/CA); 5—Atlantic Forest biome (AT); 6—Pampas biome (PA); and 7 Puerto Rico (Figure 1).

We estimated the number of transitions and transversions and the nucleotide (*π*) and haplotype diversity (*Ĥ*) (Nei, 1987). The neutrality tests Tajima's *D* (Tajima, 1989), Fu's Fs (Fu, 1997) and the mismatch distribution (Rogers & Harpending, 1992) were performed to test the neutral hypothesis and investigate the demographic events. The neutrality tests were tested for significance by generating 1,000 random samples using coalescent simulations. Hierarchical analysis of molecular variance (AMOVA) was conducted to estimate the population genetic structure within and among morphoclimatic groupings with 10,000 permutations and gamma = 0. Pairwise comparisons of the fixation index, *F*_ST_, were used to determine the genetic differences among groupings with the significance assessed by 10,000 permutations for each pairwise comparison and the gamma α value set to zero. All analyses were carried out using Arlequin v.3.5 (Excoffier & Lischer, 2010).

#### 2.2.3 Genealogical inferences

An unrooted statistical parsimony haplotype network (gene genealogies) was estimated with the default 95% plausible connection limit using the TCS 1.2.1 (Clement, Posada, & Crandall, 2000) to investigate genealogical relationships among haplotypes originating from the concatenated mtDNA sequences (COI, COII, and Cyt *b*). A second haplotype network of COI mtDNA was estimated with the dataset used in the ABC analysis (Americas, Asia, Europe and Africa) with 230 other sequences from this study, 65 from Mastrangelo et al. (2014), and 139 from Leite et al. (2014).

The Bayesian phylogenetic analysis was carried out using the 54 haplotypes (concatenated mtDNA sequences) of *H. armigera* and one mtDNA haplotype of *H. zea* as outgroup. The best substitution model of evolution selected by MrAIC v. 1.4.2 (Nylander, 2004) was HKY+I+G. Subsequently, the Bayesian phylogeny was performed in MrBAYES v3.1.2 (Ronquist & Huelsenbeck, 2003) using two simultaneous runs with four chains in each run for a total of 20 million generations. The first trees (25%) were discarded as burn‐in samples. The consensus tree was obtained with posterior probabilities > 0.50 and visualized in FigTree v.1.3.1 (Rambaut, 2009).

### 2.3 nDNA‐based structure in *H. armigera* in Brazil

Ninety‐six *H. armigera* individuals, representative of all geographical regions, were genotyped using three nDNA markers (Exon Primed Intron Crossing—EPIC): dopa decarboxylase (DDC; nDNA‐DDC‐F1/nDNA‐DDC‐R1), the ribosomal protein S6 (RpS6; nDNA‐RpS6‐F/nDNA‐RpS6‐R), and the ribosomal protein L11 (RpL11; nDNA‐RpL11‐F/nDNA‐RpL11‐R) (Tay, Behere, Heckel, Lee, & Batterham, 2008; Appendix A). Tay et al. (2013) used the same three markers to investigate samples collected in Brazil, in addition to other studies that used this approach, representing an accumulation of knowledge (Behere, Tay, Russell, Kranthi, & Batterham, 2013; Tay et al., 2008). We chose these markers because of the 12 nDNAs designed by Tay et al. (2008) specifically for *H. armigera*, in which only three had more than 10 alleles. All others had ≤four alleles and consequently low values of heterozygosity. Another option of fast‐evolving markers would be microsatellites, but several lepidopteran microsatellite markers presented amplification faults (Tay, Behere, Batterham, & Heckel, 2010), which can be softened when the primers are designed to anneal in the less variable regions of the exons, as in the case of nDNA‐EPIC (Behere et al., 2013).

The PCR was performed in a final volume of 15 μl with 25 ng of total DNA, 1.0 U of TaqDNA polymerase (Fermentas International Inc., Canada), 92 µM of dNTPs, 1.7 mM of MgCl_2_, 0.6 mg/ml of BSA, 10x TaqBuffer, and 0.23 µM of each primer. The forward primers were labeled with three different fluorescent dyes (Applied Biosystems, Foster City, CA) to evaluate size polymorphism: NED™ (yellow: DDC), VIC™ (green: RpS6), and 6‐FAM™ (blue: RpL11) (Appendix A). Negative controls were included in all amplifications to identify potential contamination. After amplification and electrophoresis, PCR products were multiplexed and characterized using a 3,500 Genetic Analyzer (Applied Biosystems, Foster City, EUA) with GeneScan™ 600 LIZ™ dye Size Standard v2.0. The result of the DNA fragment length was predicted to allele size using GeneMarker® (Version 2.4.2) (SoftGenetics, State College, PA, USA).

The inbreeding among individuals within subpopulations (*F*_IS_), pairwise *F*_ST_ matrix for all pairs of morphoclimatic regions, and observed (*H*_O_) and expected (*H*_E_) heterozygosities for each nDNA marker (and combination of them) were calculated using Arlequin v.3.5. The *F*_ST_ relations results were displayed as a heatmap using the pheatmap R package (https://cran.r-project.org/web/packages/pheatmap/index.html). Deviations from Hardy–Weinberg equilibrium (HWE) were tested in Genepop v.4.2 (Option 1) (Raymond & Rousset, 1995). *p*‐Values were obtained by the Markov Chain (MC) algorithm for each locus in each population (Guo & Thompson, 1992) using 10,000 dememorizations, 1,000 batches, and 10,000 iterations per batch to ensure a standard error <0.01. The global test across loci and locality was performed using Fisher's method once the linkage disequilibrium analyses (Slatkin, 1994) showed no linkage disequilibrium at significance level = 0.05, performed in Arlequin v.3.5 with 10,000 permutations for all pairs of loci. Allelic richness was calculated by Fstat 2.9.4 (Goudet, 1995).

### 2.4 Putative hybrids inference in Brazil

Three hundred thirty‐one *H. armigera* and 61 *H. zea* individuals, previously identified by the mtDNA COI marker, were genotyped to the Internal Transcribed Spacer 1 (ITS1) region of the nuclear ribosomal DNA (rDNA) using a *Helicoverpa* spp. forward primer (3373Ha_Hz_ITS1‐F) and two species‐specific reverse primers: for the *H. armigera* (3374Ha_ITS1‐R) and for *H. zea* (3377Hz_ITS1‐R) (Perera et al., 2015; Appendix A). The conditions for the PCR were as follows: 1.0 U of TaqDNA polymerase (Fermentas International Inc., Canada), 56 µM of dNTPs, 2.5 mM of MgCl2, 0.3 mg/ml of BSA, 10× TaqBuffer, 0.16 µM of each primer, and 25 ng of total DNA in a final volume of 25 μl.

This method produces PCR distinct product sizes between *H. armigera* (147 bp) and *H. zea* (334 bp), allowing the identification of putative hybrids when checked in agarose gel electrophoresis (Perera et al., 2015). *H. armigera*, *H. zea,* and one laboratory hybrid, previously confirmed by sequencing, were used as controls for PCR standardization and genotyping of field individuals. Furthermore, the *Helicoverpa* spp. individuals that exhibited two positive bands were re‐amplified in two separate PCRs using the primer set 3373Ha_Hz_ITS1‐F/3374Ha_ITS1‐R corresponding to the ITS1 from *H. armigera* and 3373Ha_Hz_ITS1‐F/3377Hz_ITS1‐R that corresponds to the ITS1 from *H. zea*. Once more, both amplicons were visualized on agarose gel electrophoresis, purified, sequenced, aligned, and identified following the same protocols described for the mitochondrial markers (Section 2.22.2).

## 3 RESULTS

### 3.1 Approximate Bayesian Computation

Overall, *π* and *πb* were the most informative summary statistics (also *D* for scenario 3) (Figure 2). Model c (very rapid population expansion) had the highest posterior probabilities in all tested scenarios (PP = 39.9, 41.7 and 45.1, respectively; Figure 2‐I), and then, they were grouped in one last run to compare the three scenarios together. Nonetheless, in this last independent run involving the models best fitted for each scenario (1c, 2c, and 3c), the rejection step revealed scenario 2 (44.0) as best supported by the data (Figure 2‐II). Scenario 2, model c, describes the hypothesis of South America samples originating through a founding effect whose origin populations came from Europe and had a rapid population expansion.

Observed SuSt appeared within the cloud of simulated SuSt in scenarios 2 and 3, which did not happen in scenario 1 and in the last run (Figure 2), being an outlier and suggesting an inadequate adjustment of the models. The time of divergence between the American populations and their source of introduction is very recent (around 60 generations, we believe, after its official identification), which should favor their identification through ABC analysis. However, the low PP value of the models found in all runs and the observed SuSt positioned out of the cloud of simulated SuSt in scenario 1 and in the last run may be a result of the large population size on all continents, the gene flow among populations and/or multiple introductions. Another ABC analysis performed as described above, but with 470 sequences from the Americas, also found scenario 2 with the highest PP value (data not shown). Many haplotypes are also shared between South America and Europe (Appendices B and C). Our study presents the first results obtained with this exploratory analysis, and we suggest that this is a powerful tool to boost the investigation of this recent invasion in the American continent, which can be intensely driven using a genomic approach.

### 3.2 Genetic diversity, demography, and population structure

The total haplotype and nucleotide diversity for COI, COII, and Cyt *b* concatenated genes were Hd = 0.926 ± 0.006 and *π* = 0.002 ± 0.001 (Table 1 and Appendix D). The region CE/CA shows a higher number of haplotypes (*n* = 33) and the region AM a lower number of haplotypes (*n* = 12). However, the haplotype and nucleotide diversity indicate similar diversity among Brazilian regions. The variation in sample size influenced the amount of haplotypes found in the regions (Table 1). GenBank accession numbers are on https://doi.org/10.5061/dryad.rd1570s.

**Table 1:** Estimates of haplotype and nucleotide diversity from 303 *H. armigera* based on COI, COII, and Cyt *b* concatenated mtDNA

Region	Sample size	# Haplotypes	Haplotypes	Hd	*π*
AM	16	12	H1(3); H2(2); H3; H4; H5(2); H7; H9; H10; H14; H17; H46; H47	0.958 ± 0.036	0.0026 ± 0.0015
AM/CE	22	15	H1; H3(3); H4; H5(3); H7(3); H9(2); H10; H11; H13; H16; H17; H42‐H45	0.957 ± 0.026	0.0024 ± 0.0014
CE/CA	124	33	H1(24); H2(17); H3(10); H4(12); H5(9); H6(12); H7(3); H8(5); H9(4); H10(2); H11; H12(2); H14(2); H16; H19; H20; H21(2); H22; H24‐H28; H32‐H41	0.915 ± 0.012	0.0020 ± 0.0012
CE	52	20	H1(7); H2(11); H3(4); H4(2); H5(4); H6(5); H7; H8(3); H9; H10(3); H11‐H14; H17; H18(2); H19; H22; H29; H30	0.920 ± 0.021	0.0021 ± 0.0012
AT	56	20	H1(8); H2(8); H3(8); H4(6); H5(3); H6(3); H7(2); H8; H9; H11; H12; H13(3); H15(3); H20; H23(2); H31; H51‐H54	0.927 ± 0.015	0.0022 ± 0.0013
PA	29	18	H1(3); H2(4); H3(4); H4; H5; H6(2); H7(2); H8(2); H9‐H12; H14; H15; H17; H48‐H50	0.956 ± 0.021	0.0035 ± 0.0019
Puerto Rico	4	2	H1(2); 16(2)	0.667 ± 0.204	0.0016 ± 0.0013
Total	303	54	H1(48); H2(42); H3(30); H4(23); H5(22); H6(22); H7(12); H8(11); H9(10); H10(8); H11(5)‐H14(5); H15(4)‐H17(4); H18(2)‐H23(2); H24‐H54	0.926 ± 0.006	0.0023 ± 0.0013

Note

AM/CE: Amazon Forest transition with Cerrado; AM: Amazon Forest; AT: Atlantic Forest; CE/CA: Cerrado transition with Caatinga; CE: Cerrado; PA: Pampas.

Tajima's *D* value assumed −1.6326 (*p* = 0.019), while Fu's Fs assumed −25.5408 (*p* < 0.001), and both results showed a departure from mutation‐drift equilibrium, which usually indicates a sudden demographic expansion or purifying selection (Table 2). Likewise, the same result was verified in a mismatch distribution analysis, where the observed data distribution was clearly unimodal positioning out of the expected distribution and the raggedness index was small (*r* = 0.0235), which is to be expected in a population that has experienced recent expansion (Table 2).

**Table 2:** Neutrality test statistics and mismatch distribution analysis from 303 *H. armigera* based on COI, COII, and Cyt *b* concatenated mtDNA

Region	Sample size (*n*)	Tajima's *D* (*p*‐value)	Fu's Fs (*p*‐value)	*τ* (*SD*_95%_)	SSD (*p*‐value)	*r* (*p*‐value)
AM	16	−1.573 (0.04)	−5.000 (0.00)	3.5 (2.15–4.67)	0.0102 (0.18)	0.0308 (0.37)
AM/CE	22	−1.502 (0.05)	−7.181 (0.00)	3.1 (2.47–6.45)	0.0140 (0.04)	0.0439 (0.10)
CE/CA	124	−1.642 (0.02)	−18.952 (0.00)	3.1 (1.79–4.25)	0.0030 (0.09)	0.0236 (0.40)
CE	52	−1.836 (0.01)	−8.345 (0.00)	3.2 (2.93–3.26)	0.0170 (0.00)	0.0535 (0.00)
AT	56	−1.559 (0.03)	−7.055 (0.01)	3.8 (2.27–5.46)	0.0045 (0.12)	0.0210 (0.38)
PA	29	−1.020 (0.16)	−6.037 (0.01)	3.9 (2.04–5.10)	0.0195 (0.00)	0.0315 (0.09)
Puerto Rico	4	2.080 (0.98)	2.719 (0.85)	4.8 (1.92–57.79)	0.3700 (0.02)	1.0000 (0.16)
Total	303	−1.633 (0.02)	−25.541 (0.00)	3.5 (1.96–4.58)	0.0045 (0.02)	0.0235 (0.21)

Note

AM/CE: Amazon Forest transition with Cerrado; AM: Amazon Forest; AT: Atlantic Forest; CE/CA: Cerrado transition with Caatinga; CE: Cerrado; PA: Pampas.

Additionally, little evidence of subdivision into genetically distinct populations was found by AMOVA, with a variation of 92.46% occurring within sites' level and only 7.54% among sites' level also indicating genetic homogeneity Ɵ_ST_ = 0.0754 and *p*‐value = 0.0074) (Table 3). For three hierarchical levels, where the differentiation was also tested among the morphoclimatic regions, the results were similar, with −0.67% of variation occurring among regions' level and 8.08% among sites within regions' level (Table 3).

**Table 3:** Analysis of molecular variance (AMOVA) from 303 *H. armigera* based on COI, COII, and Cyt *b* concatenated mtDNA

Source of variation	*df*	Variance components	Percentage variance	Fixation indices
Among sites	68	0.141	7.54	Φ_ST_ = 0.075
Within sites	234	1.733	92.46	
Total	302	1.875		
Among regions	6	−0.012	−0.67	Φ_CT_ = −0.007
Among sites within regions	62	0.151	8.08	Φ_SC_ = 0.080
Within sites	234	1.733	92.59	Φ_ST_ = 0.074
Total	302	1.872		

### 3.3 Genealogic inferences

**Figure 3:**
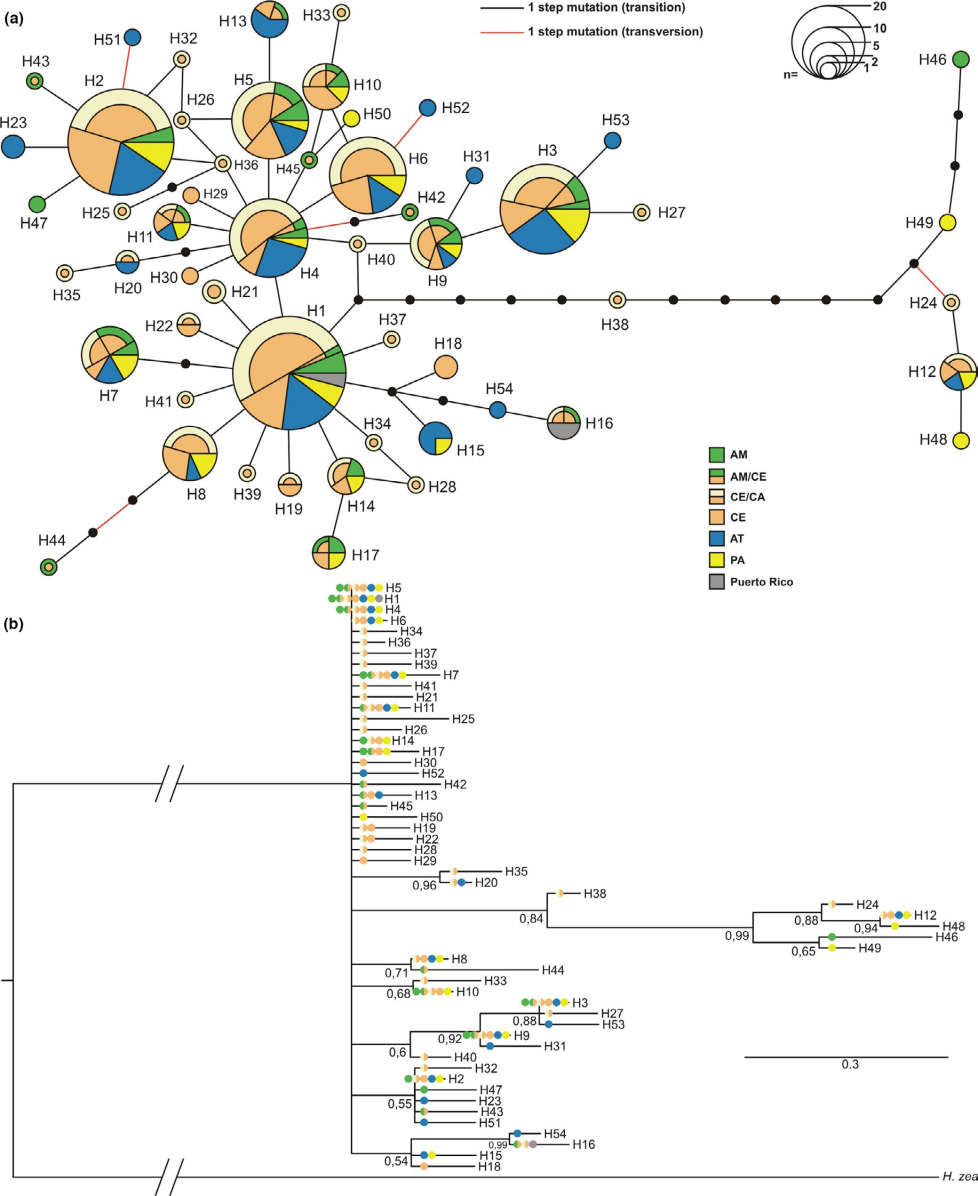
(a) Parsimony haplotype network of concatenated 1,646 bp‐long mtDNA from 303 *H. armigera* sampled in South America and Puerto Rico. Circles represent different haplotypes nominated in ascending order from most frequent to least frequent (H1‐H54). Circles area is proportional to the number of individuals carrying this haplotype as shown by the scale at the top. Colors in the circles indicate the proportional distribution of the morphoclimatic regions in each haplotype, small black dots represent presumed, but unsampled haplotypes and the connection lines are proportional to number of mutational steps between any two different haplotypes. AM: Amazon Forest; AM/CE: Amazon Forest transition with Cerrado; AT: Atlantic Forest; CE/CA: Cerrado transition with Caatinga; CE: Cerrado; PA: Pampas. (b) Phylogram of the Bayesian topology consensus tree based on the same COI mtDNA of 303 *H. armigera* sequences plus 12 *H. zea* sequences used here as outgroup. Numbers at forks represent the Posterior Probability and, like in the haplotype network, the circles colored indicate the same morphoclimatic regions where each haplotype was found

The 54 haplotypes showed a complex and diversified topology in the haplotype network (Figure 3a). Six haplotypes in the high frequency range (H1–H6) are centrally located in the network and accompanied by a large number of private haplotypes (*n* = 31; 57.4%). A haplogroup separated for 12 mutation steps is represented by haplotypes H49, H48, H46, H24, and H12. The Bayesian phylogenetic analyses show short branch and low supported nods (Figure 3b). Additionally, the phylogeny confirmed the presence of a more recent haplogroup supported for a posteriori probability of 0.84. Both, the haplotype network and the Bayesian tree do not suggest predominance of specific haplotypes among sites or morphoclimatic regions (Figure 3). The global network for the COI mtDNA showed 57 haplotypes with a complex genealogic relationship, which has two central and frequent haplotypes and others haplotypes surrounding them. The five sequences of Puerto Rico were organized into two haplotypes, the H2 shared with all regions of the world (includes Brazil) and a haplotype H9 shared only with the northwestern Brazil (Appendices B and C).

### 3.4 nDNA‐PCR‐based structure in *H. armigera*

A total of 92 samples were successfully predicted to allele size for all three nDNA‐PCR markers (data are available on Dryad: https://doi.org/10.5061/dryad.rd1570s). The remaining four samples, which had unspecific amplification for any nDNA marker, were discarded from the analysis. From those samples, 56 alleles were found in total for the three markers (Table 4). DDC and RpS6 had a large number of alleles (ranging from 170 to 354 bp and from 215 to 290 bp, respectively), higher values of allelic richness (from 3.0 to 6.3) and high expected (*H*_E_) and observed (*H*_O_) heterozygosities (0.913, 0.761 and 0.873, 0.760, respectively). Most of the localities were in HWE, except DDC marker from CE/CA and RpS6 marker from AM/CE, CE, and AT. All of them had a *p*‐value < 0.05. When all samples were joined and treated as one locality, both DDC and RpS6 were not in HWE. On the other hand, when the three markers were treated together, AM/CE, CE/CA, and CE were significantly out of HWE. Except for RpL11 marker, the *F*_IS_ values in each locality ranged widely from −0.5 (RpS6 in Puerto Rico) to 0.493 (RpS6 in AM/CE). The AMOVA showed that only 1.31% of variation occurred among sites' level, and 98.69% within sites' level (global *F*_ST_ = 0.0130 and *p*‐value = 0.1233). *F* statistics (Weir & Cockerham, 1984) were *F*_ST_ = 0.0075 (*p* > 0.9), *F*_IS_ = 0.1343 (*p* < 0.001), and *F*_IT_ = 0.1408 (*p* < 0.001), respectively.

**Table 4:** Estimates of population statistics based on DDC, RpL11, and RpS6 nDNA markers from 92 *H. armigera* individuals

Region	*N*	DDC					RpL11					RpS6					*H*_E_ Mean (*SD*)	*H*_O_ Mean (*SD*)	HW‐*p*(all loci)
		Allelic Richness	*H*_E_	*H*_O_	HW‐*p*	*F*_IS_	Allelic Richness	*H*_E_	*H*_O_	HW‐*p*	*F*_IS_	Allelic Richness	*H*_E_	*H*_O_	HW‐*p*	*F*_IS_			
AM	8	5.528	0.892	0.875	0.867	0.020	1.500	0.125	0.125	—	—	5.090	0.867	1.000	0.263	−0.167	0.628 (0.436)	0.667 (0.473)	0.566
AM/CE	7	5.263	0.824	0.571	0.051	0.324	1.000	—	—	—	—	4.531	0.813	0.429	0.026	0.493	0.546 (0.473)	0.500 (0.101)	0.010
CE/CA	27	5.888	0.906	0.630	0.001	0.309	1.296	0.073	0.074	1.000	−0.010	5.933	0.906	0.778	0.128	0.143	0.628 (0.481)	0.494 (0.371)	0.007
CE	15	6.327	0.931	0.867	0.622	0.071	1.533	0.131	0.133	1.000	−0.018	5.866	0.906	0.667	0.000	0.279	0.656 (0.455)	0.556 (0.379)	High. sign.
AT	15	5.825	0.901	0.800	0.340	0.116	1.736	0.191	0.200	1.000	−0.050	4.600	0.816	0.733	0.022	0.105	0.636 (0.388)	0.578 (0.329)	0.134
PA	16	5.793	0.895	0.938	0.782	−0.049	1.250	0.063	0.063	—	—	5.767	0.897	0.813	0.231	0.097	0.618 (0.481)	0.604 (0.473)	0.490
Puerto Rico	4	3.000	0.714	0.500	0.086	0.333	1.000	—	—	—	—	3.000	0.714	1.000	0.543	−0.500	0.476 (0.412)	0.750 (0.354)	0.190
Total	92	6.060	0.913	0.761	0.013	0.167	1.380	0.095	0.098	1.000	−0.027	5.412	0.873	0.760	High. sign.	0.129	0.627 (0.461)	0.540 (0.382)	High. sign.

Notes

Expected (*H*_E_) and observed (*H*_O_) heterozygosities, Hardy–Weinberg equilibrium *p*‐values (HW‐*p*), inbreeding in absolute values for localities per polymorphic locus (*F*_IS_).

AM/CE: Amazon Forest transition with Cerrado; AM: Amazon Forest; AT: Atlantic Forest; CE/CA: Cerrado transition with Caatinga; CE: Cerrado; PA: Pampas.

**Figure 4:**
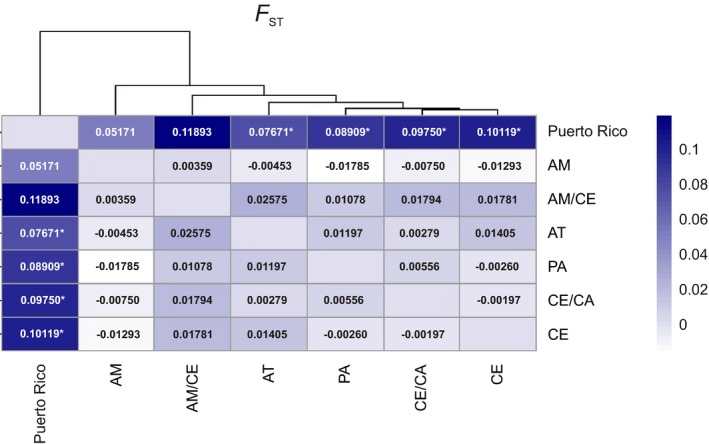
Heatmap of pairwise *F*_ST_ distances ranging from low (white) to high (blue) between morphoclimatic regions estimated from nDNA markers of 92 *H. armigera* samples data. AM: Amazon Forest; AM/CE: Amazon Forest transition with Cerrado; AT: Atlantic Forest; CE/CA: Cerrado transition with Caatinga; CE: Cerrado; PA: Pampas. *p‐value < 0.05

Pairwise *F*_ST_ values varied from −0.018 to 0.119 (Figure 4), and four comparisons involving Puerto Rico (with PA, AT, CE and CE/CA) had a *p* < 0.05, indicating population differentiation. The other two comparisons with Puerto Rico (AM and AM/CE) had a relatively high *F*_ST_ value but were not significant. All other comparisons had low *F*_ST_ values (−0.018 to 0.026) and were not significant showing greater homogeneity. The data also show, even subtly, that Puerto Rico is closer to the AM and AM/CE samples (also geographically closer) than to other morphoclimatic regions.

### 3.5 Putative hybrids detection

Of 392 *Helicoverpa*spp. individuals tested, twelve samples (all of them initially identified as *H. armigera*by mtDNA markers) exhibited two bands on agarose gel electrophoresis (~147 bp and ~334 bp). Thus, they could be hybrids between *H. armigera* and *H. zea* because these species‐specific sequences were found in a single individual. After the sequencing, alignment, and GenBank conference, only six individuals had their 147 bp‐long amplicons matched >98% with ITS1 from *H. armigera* and its 334 bp‐long amplicons matched > 98% with ITS1 from *H. zea* (Appendices E and F). Moreover, these sequences match the correspondent ones from a hybrid reared in laboratory. These individuals were classified as putative hybrids and originated from AM/CE (two individuals), CE/CA, AM, PA, and CE (one individual each) (GenBank accession numbers: MG893730‐MG893735). Both bands from another six samples only matched ITS1 from *H. armigera*, but in different sizes, which suggests a nonspecificity of the primers, mainly 3377Hz_ITS1‐R.

## 4 DISCUSSION

### 4.1 Inferring South America *H. armigera* origin

ABC is a powerful approach to investigate complex demographic events and biological invasion source that occurred in the past (Camargo et al., 2012; Tsai & Carstens, 2013). Here, we used ABC to investigate the *H. armigera* invasion source in the South American continent and it suggests Europe as its origin. After the rapid population expansion in South America, *H. armigera* would have dispersed from South America to the Caribbean region. European origin of the species in Brazil is supported by reports of human‐mediated dispersion via international trade of agricultural products between the Americas and Europe over the past 500 years, trade that has intensified over the last 100 years and much more over the last three decades of globalization (Barbosa et al., 2015; Biondi, Guedes, Wan, & Desneux, 2018; di Castri, 1989; Grapputo, Boman, Lindstroem, Lyytinen, & Mappes, 2005; Oliveira, Corrêa, Souza, Guedes, & Oliveira, 2013; Paini et al., 2016; Weintraub et al., 2017).

We found two haplotypes in Puerto Rico for the COI (Appendices B and C). One of them is the second most frequent haplotype in its world distribution. The other has only been found in the northwestern region of Brazil. Thus, we believe these are clues suggesting that individuals found in Puerto Rico should have originated in South America, through a process of population geographical expansion after the initial infestation, despite the high genetic differentiation found using nDNA markers.

Common mtDNA haplotypes are shared among the Americas, Europe, Africa, and Asia, including the sharing of alleles associated with pyrethroid resistance (Durigan et al., 2017; Tay et al., 2017). Furthermore, *H. armigera* populations show weak genetic structure and high gene flow in the Old World and Australia (Anderson et al., 2016; Behere et al., 2007; Endersby, Hoffmann, McKechnie, & Weeks, 2007; Li et al., 2011; Nibouche, Bues, Toubon, & Poitout, 1998; Tay et al., 2008; Vijaykumar, Krishnareddy, Kuruvinashetti, & Patil, 2008; Weeks et al., 2010). The high diversity and weak genetic structure in the Old World make it difficult to describe with accuracy the geographical origin, routes, and the number of invasion events of *H. armigera* to Brazil. However, they also reduce the impact of this information because there is a wide transference of alleles among geographical sites in the Old World (Anderson et al., 2016; Behere et al., 2007; Nibouche et al., 1998; Rasool et al., 2014; Stokes, Mckechnie, & Forrester, 1997). Finally, there is consensus that the invasion of South America by *H. armigera* was not originated from Oceania (Anderson et al., 2016; Durigan et al., 2017; Tay et al., 2017).

### 4.2 High genetic diversity and population expansion of *H. armigera*

Unexpected high genetic diversity was confirmed in *H. armigera* from Brazil what is not common in a recent invasive species (Handley et al., 2011). High population diversity and population admixture of *H. armigera* in the Old World are probably explanations for the high diversity of *H. armigera* in Brazil (Behere et al., 2007; Genton, Shykoff, & Giraud, 2005; Lombaert et al., 2010; Rius & Darling, 2014; Sosa‐Gomez et al., 2016). Mitochondrial and nuclear DNA diversity indices are similar among regions, revealing a wide distribution of *H. armigera* diversity in all Brazilian territory. The higher number of haplotypes found in CE/CA and CE morphoclimatic regions can be explained by a sampling disequilibrium of specimens among these regions.

The high number of private haplotypes (57%) separated for a single mutation step suggests that *H. armigera* populations are in demographic expansion in Brazil, which is also confirmed by the neutrality tests and the unimodal mismatch distribution analysis. Population expansion is a common fact in invasive species (Cesari et al., 2017; Lee, 2002; Sakai et al., 2001). However, *H. armigera* population expansion may also be justified by evolutionary forces resulting from agricultural management that promotes multiple events of colonization in the areas. This approach may be relevant to other insect pests since demographic expansion is commonly reported in native agricultural pests in Brazil (Albernaz, Silva‐Brandão, Fresia, Cônsoli, & Omoto, 2012; Fresia, Lyra, Coronado, & Azeredo‐Espin, 2011; Silva‐Brandão, Santos, Cônsoli, & Omoto, 2015; Soares, Cordeiro, Santos, Omoto, & Correa, 2018).

The genealogic relation among all 54 mtDNA haplotypes built a complex haplotype network, where the most frequent haplotypes are spread in all morphoclimatic regions of Brazil. The haplotype network does not show a star‐like standard, frequently reported in organisms of recent invasion, reinforcing the hypothesis of multiple (or a huge) invasion events in South America. A more recent haplogroup was identified in the analysis, probably associated with a vicariance/sympatric event among *H. armigera* populations on Old World continents. This haplogroup divergence event among haplotypes is not expected for *H. armigera* in South America due to its recent invasion, population expansion, and absence of genetic structure on the continent (Leite, Corrêa, et al., 2017; Tay et al., 2013, 2017).

### 4.3 No spatial structure of *H. armigera* in South America

nDNA‐PCR markers yielded concordant results with mtDNA sequences data revealing lack of population structure of *H. armigera* in the South America. The lack of genetic structure of *H. armigera* is consistent with traits of (a) a pest of recent invasion, (b) huge dispersion capacity of the moths carried by wind, and (c) a high polyphagia associated with uninterrupted and diversified crops during the entire year, stimulating a high reproductive rate of *H. armigera* in South America (Fitt, 1989; Hardwick, 1965; Nibouche et al., 1998). The genetic variation is primarily within populations with recent population expansion and multidirectional dispersion among sites and morphoclimatic regions. The weak geographical population structure detected at the regional level in South American populations of *H. armigera* was also described at continental and intercontinental levels in the Old World (Anderson et al., 2016; Behere et al., 2013; Nibouche et al., 1998; Scott et al., 2005; Song et al., 2018). High population admixture has serious implications for pest management, since alleles may spread to wide areas and promote an increase of genetic diversity and fitness in local populations of *H. armigera* (Fraimout et al., 2017; Lombaert et al., 2010; Rius & Darling, 2014; Seymour et al., 2016).

High genetic differentiation of *H. armigera* from South America and Puerto Rico show a limited gene flow between South and Central America. Low gene flow of Noctuidae pests between South and Central and North America was reported to *Spodoptera frugiperda* and *H. zea* (Leite, Corrêa, et al., 2017; Nagoshi et al., 2017). It is probably associated with contrary wind patterns between the South and North American continents that limit long‐distance dispersion of Noctuidae moths, providing few opportunities for gene flow. Of the 450 *H. armigera* male individuals trapped at 12 sites in Puerto Rico from 2016 to 2017, only five individuals were identified as *H. armigera*. This reinforces the low capacity of locomotion of *H. armigera* between South and North America and helps to explain the low population density and the nonestablishment of *H. armigera* populations in Central and North America even in the absence of wide ocean barriers separating the continent.

### 4.4 Presence of Helicoverpa putative hybrids in Brazil

We found a low number of putative hybrids between *H. armigera* and *H. zea* Brazilian strains under natural conditions. This result agrees with the previous studies that suggest low hibridization between *H. armigera* and *H. zea* in Brazil (Anderson et al., 2018, 2016; Leite, Pereira, et al., 2017. However, Brazilian agroecosystems show abrupt changes of cultivated area size, crop species, and pest management strategies (e.g., *Bt* crop events), which may rapidly modify the hybridization dynamic between *H. armigera* and *H. zea*. Thus, a constant monitoring of *H. armigera* and *H. zea* populations is recommended, since introgressions of adaptive genes/alleles between *H. armigera* and *H. zea* may have implications for the population management of these pests.

In conclusion, we found demographic signals of European origin of *H. armigera* in South America what could be supported by the history of invasion events between both continents. Like the European colonization after the great navigations, the invading *H. armigera* populations have established successfully on the South American continent, and their expansion north continues. The highly diverse genetic structure, recent expansion population, and multidirectional gene flow, plus the presence of putative hybrids, assumes a scenario of uncertainties for pest management of *H. armigera*. Alleles previously selected may spread long distances and, eventually, be transferred between the species, promoting a fitness increase in *Helicoverpa* local populations and the rapid evolution of resistance to pesticides and *Bt* crops in South America (Chakroun et al., 2016; Downes, Walsh, & Tay, 2016; Han et al., 2015; Joußen & Heckel, 2016).

## CONFLICT OF INTEREST

We have no competing financial interests.

## AUTHORS' CONTRIBUTION

RMG, TM, JCVR, DFP, and AMLAE conceived and designed the study. RMG collected the data. ASC, CO, and AMLAE provided reagents and analytical tools. RMG, TM, and ASC analyzed the data. RMG, TM, and ASC wrote the manuscript. All authors read, corrected, and approved the manuscript.

## Data Availability

All COI, COII, Cyt *b,* and ITS1 sequences used in this study were deposited in the NCBI database under accession number (KT828763–KT829449 and MG893581–MG893843). The detailed information about the sequences and all scripts used in ABC analysis are available at the public Dryad Digital Repository: https://doi.org/10.5061/dryad.rd1570s.

## APPENDIX A

### PRIMER SEQUENCE AND PCR CONDITIONS

**Table:**

Primers	Primer sequence (5′ ‐> 3′)	PCR product size (bp)	PCR conditions (ºC/s)				
			Initial Denat.	35 cycles			Final Ext.
				Denat	Annel	Exten	
*COI*F (COI)	ATTCAACCAATCATAAAGATATTGG	658	94/180	94/30	45/30	70/90	70/420
*COI*R (COI)	TAAACTTCTGGATGTCCAAAAAATCA						
A‐tLEU (COII)	ATGGCAGATTAGTGCAATGG	687	94/180	94/30	55/30	72/60	72/480
B‐tLYS (COII)	GTTTAAGAGACCAGTACTTG						
Harm_CB_J (Cyt *b*)	CGTACCCTTCATGCTAACGGAGC	557	94/180	94/30	55/30	72/60	72/480
Harm_CB_N (Cyt *b*)	GGAGTTACTAATGGATTTGCTGGG						
3373Ha_Hz_ITS1‐F	GAGGAAGTAAAAGTCGTAACAAGGTTTCC	147(*Ha*) e 334(*Hz*)	95/180	95/30	60/30	72/60	72/300
3374Ha_ITS1‐R	CGTTCGACTCTGTGTCCTCTAGTGG						
3377Hz_ITS1‐R	TTGATTGTTAACGAACGCGCCG						
nDNA‐DDC‐F1	(NED)CGTCGAAGGCGCAAGATGATGTT	206	95/180	95/60	53/60	72/60	72/600
nDNA‐DDC‐R1	GAGGAAGATATCCGCAATGGATTG						
nDNA‐RpS6‐F	(VIC)AGCARGGCTTCCCSATGAARCAG	274	95/180	95/60	55/60	72/60	72/600
nDNA‐RpS6‐R	CTTTGACATCARCARACGAACACG						
nDNA‐RpL11‐F	(6‐FAM)CTTTTTGAAAAGTTGTAATCATGG	287	95/180	95/60	60/60	72/60	72/600
nDNA‐RpL11‐R	CAACGCRGGCGGTKGTACACG						

Notes

Annel = annealing; Denat = denaturing; Exten = extension; Final Ext. = final extension; Initial Denat. = initial denaturing.

Fluorescent‐labeled forward primers. The NED™, VIC™ and 6‐FAM™ dye labels (Thermo Fisher Scientific Inc., Waltham, EUA) used are shown in the corresponding primer sequence at its 5′ end. *Ha* = *H. armigera*, *Hz* = *H. zea*.

## APPENDIX B

Parsimony haplotype network of COI 626 bp‐long mtDNA from 526 *H. armigera* sampled in the Americas (*n* = 470), Asia (*n* = 40), Europe (*n* = 11), and Africa (*n* = 5). Circles represent different haplotypes nominated in ascending order from most frequent to least frequent (1–57). Circles area is proportional to the number of individuals carrying this haplotype as shown by the scale at the top‐right. Colors in the circles indicate the proportional distribution of the continents in each haplotype, small black dots represent presumed, but unsampled haplotypes and the number of connection lines are proportional to number of mutational steps between any two different haplotypes. The five sequences found in Puerto Rico are indicated in the two corresponding haplotypes (2 and 9).

## APPENDIX C

Sharing of haplotypes based on cytochrome *c* oxidase subunit I (COI) among South America, Central America, Asia, Europe, and Africa continents.

**Table:**

Haplotype (*n*)	Continent (*n*)	GenBank accession numbers	References
1 (174)	South America (168) Asia (4) Europe (2)	AB620128, EU768935, FN907996, FN907997, GQ892847, GQ995232, KM274936, KM274939, KM274940, KM274958–KM274960, KM274964, KM274966–KM274969, KM274972, KM274975, KM274980, KM274981, KM274988, KM274989, KM274994, KM275046, KM275051, KM275052, KM275070, KM275073, KM275075, KM275078, KM275086, KM275088, KM275098, KM275099, KM275104, KM275106, KM275107, KM275108, KM275110, KM275111, KM275127, KM275134, KM275138, KM275140, KM275147, KM275148, KM275154, KM275156, KT828802–KT828827, KT828851–KT828889, KT828922–KT828929, KT828941–KT828943, KT828952, KT828953, KT828965, KT828966, KT828969, KT828971, KT828974, KT828975, KT828979, KT828985, KT828990, MG893742, MG893743, MG893746, MG893748, MG893750, MG893751, MG893754–MG893763, MG893778, MG893791, MG893792, MG893797, MG893798, MG893801, MG893803, MG893804, MG893812, MG893814, MG893815, MG893817–MG893819, MG893825, MG893831, MG893835, MG893836, MG893839, MG893840, MG893842, MG893752	In this work, Leite et al. (2014), Li et al. (2011), Unpublished
2 (151)	Central America (3) South America (130) Asia (11) Europe (4) Africa (3)	FN907995, FN908000, FN908003, FN908005, FN908011, FN908013–FN908015, FN908018, GQ892843, GQ892848, GQ892855, GQ995233–GQ995236, JX532104, KJ460247, KM274938, KM274943, KM274945, KM274946, KM274949, KM274950, KM274953, KM274961, KM274965, KM274970, KM274973, KM274974, KM274984–KM274987, KM274990, KM274992, KM274993, KM274995, KM274996, KM275040, KM275076, KM275081, KM275083, KM275084, KM275090, KM275091, KM275100, KM275103, KM275105, KM275152, KM275158, KT828763–KT828921, KT828937–KT828940, KT828947–KT828949, KT828958, KT828959, KT828968, KT828973, KT828976, KT828982, KT828991, MG893744, MG893745, MG893747, MG893753, MG893767, MG893777, MG893780, MG893782, MG893786, MG893788, MG893790, MG893795, MG893796, MG893800, MG893802, MG893805, MG893807, MG893821, MG893823, MG893824, MG893826, MG893827, MG893829, MG893834, MG893838, MG893843, MG893785	In this work, Leite et al. (2014), Li et al. (2011), Unpublished
3 (60)	South America (58) Europe (2)	FN908001, GU686757, KM274947, KM274957, KM274983, KM275039, KM275043, KM275047–KM275050, KM275072, KM275080, KM275085, KM275087, KM275089, KM275092, KM275097, KM275109, KM275137, KM275149, KM275150, KM275155, KM275157, KT828828‐KT828850, KT828967, KT828981, KT828989, MG893749, MG893776, MG893794, MG893799, MG893806, MG893809, MG893813, MG893822, MG893833, MG893841	In this work, Leite et al. (2014), Unpublished
4 (31)	South America (30) Asia (1)	GQ995242, KM274944, KM275202, KM275204–KM275206, KT828890–KT828901, MG893764–MG893766, MG893768–MG893772, MG893779, MG893781, MG893787, MG893816, MG893830	In this work, Leite et al. (2014), Li et al. (2011)
5 (17)	South America (14) Asia (1) Europe (1) Africa (1)	FN908002, FN908017, GQ892854, KM274963, KM274979, KM275074, KM275129, KM275130, KM275132, KT828944–KT828946, KT828964, KT828987, MG893793, MG893810, MG893811	In this work, Leite et al. (2014), Li et al. (2011), Unpublished
6 (15)	South America (15)	KM274971, KM275044, KM275071, KM275079, KT828930–KT828936, KT828972, MG893789, MG893832, MG893837	In this work, Leite et al. (2014)
7 (8)	South America (2) Asia (5) Europe (1)	FN907999, GQ892842, GQ995237, GQ995238, HM854928, HM854932, KM275112, KT828984	In this work, Leite et al. (2014), Li et al. (2011), Unpublished
8 (5)	Asia (5)	GQ892840, GQ892844, GQ892845, GQ892851, GQ892852	Li et al. (2011)
9 (5)	Central America (2) South America (3)	KT828956, KT828957, MG893783, MG893784, MG893820	In this work
10 (5)	South America (5)	KT828954, KT828955, MG893773–MG893775	In this work
11 (3)	South America (3)	KT828950, KT828951, MG893828	In this work
12 (3)	South America (2) Asia (1)	FN908016, KT828962, KT828963	In this work, Unpublished
13 (2)	South America (2)	KT828960, KT828961	In this work
14 (2)	South America (2)	KM274982, KM275153	Leite et al. (2014)
15 (2)	South America (2)	KM275128, KM275139	Leite et al. (2014)
16 (2)	Asia (1) Europe (1)	FN907998, HM854930	Unpublished
17 (1)	South America (1)	KM274937	Leite et al. (2014)
18 (1)	South America (1)	KM274941	Leite et al. (2014)
19 (1)	South America (1)	KM274948	Leite et al. (2014)
20 (1)	South America (1)	KM274951	Leite et al. (2014)
21 (1)	South America (1)	KM274952	Leite et al. (2014)
22 (1)	South America (1)	KM274962	Leite et al. (2014)
23 (1)	South America (1)	KM274991	Leite et al. (2014)
24 (1)	South America (1)	KM275038	Leite et al. (2014)
25 (1)	South America (1)	KM275041	Leite et al. (2014)
26 (1)	South America (1)	KM275042	Leite et al. (2014)
27 (1)	South America (1)	KM275045	Leite et al. (2014)
28 (1)	South America (1)	KM275077	Leite et al. (2014)
29 (1)	South America (1)	KM275082	Leite et al. (2014)
30 (1)	South America (1)	KM275101	Leite et al. (2014)
31 (1)	South America (1)	KM275102	Leite et al. (2014)
32 (1)	South America (1)	KM275131	Leite et al. (2014)
33 (1)	South America (1)	KM275133	Leite et al. (2014)
34 (1)	South America (1)	KM275135	Leite et al. (2014)
35 (1)	South America (1)	KM275136	Leite et al. (2014)
36 (1)	South America (1)	KM275151	Leite et al. (2014)
37 (1)	South America (1)	KM275203	Leite et al. (2014)
38 (1)	South America (1)	KT828970	In this work
39 (1)	South America (1)	KT828977	In this work
40 (1)	South America (1)	KT828978	In this work
41 (1)	South America (1)	KT828980	In this work
42 (1)	South America (1)	KT828983	In this work
43 (1)	South America (1)	KT828986	In this work
44 (1)	South America (1)	MG893808	In this work
45 (1)	South America (1)	KT828988	In this work
46 (1)	Asia (1)	GQ892846	Li et al. (2011)
47 (1)	Asia (1)	GQ892849	Li et al. (2011)
48 (1)	Asia (1)	GQ892850	Li et al. (2011)
49 (1)	Asia (1)	GQ892853	Li et al. (2011)
50 (1)	Asia (1)	GQ995239	Li et al. (2011)
51 (1)	Asia (1)	GQ995240	Li et al. (2011)
52 (1)	Asia (1)	GQ995241	Li et al. (2011)
53 (1)	Asia (1)	GQ995243	Li et al. (2011)
54 (1)	Asia (1)	GQ995244	Li et al. (2011)
55 (1)	Asia (1)	HM854929	Unpublished
56 (1)	Asia (1)	HM854931	Unpublished
57 (1)	Africa (1)	FN908006	Unpublished

## APPENDIX D

Estimates of haplotype and nucleotide diversity from 303 *H. armigera* based on COI, COII, and Cyt *b* concatenated mtDNA separated by collection sites.

**Table:**

Region	Locality	*N*	Nhp	Hd	*π*	Transitions/transversions
AM	Santarém/PA	1	1	1.000 ± 0.000	0.0000 ± 0.0000	0/0
AM	Santarém/PA	1	1	1.000 ± 0.000	0.0000 ± 0.0000	0/0
AM	Boa Vista/RR	11	8	0.927 ± 0.067	0.0019 ± 0.0012	13/1
AM	Bonfim/RR	3	2	0.667 ± 0.314	0.0045 ± 0.0036	10/1
AM/CE	Vilhena/RO	2	1	0.000 ± 0.000	0.0000 ± 0.0000	0/0
AM/CE	Itapuã D'Oeste/RO	2	2	1.000 ± 0.500	0.0030 ± 0.0033	5/0
AM/CE	Porto Velho/RO	3	3	1.000 ± 0.272	0.0024 ± 0.0021	4/2
AM/CE	Seringueiras/RO	1	1	1.000 ± 0.000	0.0000 ± 0.0000	0/0
AM/CE	Rolim de Moura/RO	2	2	1.000 ± 0.500	0.0024 ± 0.0027	4/0
AM/CE	Ariquemes/RO	1	1	1.000 ± 0.000	0.0000 ± 0.0000	0/0
AM/CE	Parecis/RO	1	1	1.000 ± 0.000	0.0000 ± 0.0000	0/0
AM/CE	Cerejeiras/RO	1	1	1.000 ± 0.000	0.0000 ± 0.0000	0/0
AM/CE	Chupinguaia/RO	3	3	1.000 ± 0.272	0.0024 ± 0.0021	6/0
AM/CE	Espigão D'Oeste/RO	3	3	1.000 ± 0.272	0.0020 ± 0.0018	5/0
AM/CE	Alto Alegre dos Parecis/RO	2	2	1.000 ± 0.500	0.0043 ± 0.0045	7/0
AM/CE	Pimenteiras D'Oeste/RO	1	1	1.000 ± 0.000	0.0000 ± 0.0000	0/0
CE/CA	Cruz das Almas/BA	2	2	1.000 ± 0.500	0.0024 ± 0.0027	4/0
CE/CA	Balsas/MA	28	16	0.913 ± 0.038	0.0023 ± 0.0013	31/2
CE/CA	Riachão das Neves/BA	5	4	0.900 ± 0.161	0.0013 ± 0.0010	5/0
CE/CA	Luis Eduardo Magalhães/BA	2	2	1.000 ± 0.500	0.0091 ± 0.0094	14/1
CE/CA	Bom Jesus/PI	11	6	0.873 ± 0.071	0.0015 ± 0.0010	8/0
CE/CA	Luis Eduardo Magalhães/BA	1	1	1.000 ± 0.000	0.0000 ± 0.0000	0/0
CE/CA	Luis Eduardo Magalhães/BA	2	2	1.000 ± 0.500	0.0024 ± 0.0027	4/0
CE/CA	Luis Eduardo Magalhães/BA	1	1	1.000 ± 0.000	0.0000 ± 0.0000	0/0
CE/CA	Luis Eduardo Magalhães/BA	1	1	1.000 ± 0.000	0.0000 ± 0.0000	0/0
CE/CA	Luis Eduardo Magalhães/BA	1	1	1.000 ± 0.000	0.0000 ± 0.0000	0/0
CE/CA	Correntina/BA	1	1	1.000 ± 0.000	0.0000 ± 0.0000	0/0
CE/CA	Correntina/BA	2	2	1.000 ± 0.500	0.0030 ± 0.0033	5/0
CE/CA	Correntina/BA	1	1	1.000 ± 0.000	0.0000 ± 0.0000	0/0
CE/CA	Rosário/BA	2	2	1.000 ± 0.500	0.0085 ± 0.0088	13/1
CE/CA	Uruçuí/PI	39	16	0.906 ± 0.027	0.0016 ± 0.0010	20/0
CE/CA	Luis Eduardo Magalhães/BA	19	3	0.573 ± 0.061	0.0010 ± 0.0007	4/0
CE/CA	Palmas/TO	6	4	0.800 ± 0.172	0.0010 ± 0.0008	5/0
CE	Iraí de Minas/MG	1	1	1.000 ± 0.000	0.0000 ± 0.0000	0/0
CE	Monte Carmelo/MG	12	8	0.924 ± 0.058	0.0020 ± 0.0012	11/0
CE	Nova Ponte/MG	4	4	1.000 ± 0.177	0.0022 ± 0.0017	7/0
CE	Costa Rica/MS	4	4	1.000 ± 0.177	0.0018 ± 0.0014	6/0
CE	Paraíso das Águas/MS	4	4	1.000 ± 0.177	0.0054 ± 0.0038	16/1
CE	Rondonópolis/MT	5	5	1.000 ± 0.127	0.0017 ± 0.0013	7/0
CE	Palminópolis/GO	1	1	1.000 ± 0.000	0.0000 ± 0.0000	0/0
CE	Palmeiras de Goiás/GO	1	1	1.000 ± 0.000	0.0000 ± 0.0000	0/0
CE	Palmeiras de Goiás/GO	1	1	1.000 ± 0.000	0.0000 ± 0.0000	0/0
CE	Morrinhos/GO	2	2	1.000 ± 0.500	0.0024 ± 0.0027	4/0
CE	Morrinhos/GO	1	1	1.000 ± 0.000	0.0000 ± 0.0000	0/0
CE	Morrinhos/GO	3	3	1.000 ± 0.272	0.0016 ± 0.0015	4/0
CE	Morrinhos/GO	1	1	1.000 ± 0.000	0.0000 ± 0.0000	0/0
CE	Morrinhos/GO	5	5	1.000 ± 0.127	0.0018 ± 0.0013	6/1
CE	Santo Antônio de Goiás/GO	1	1	1.000 ± 0.000	0.0000 ± 0.0000	0/0
CE	Primavera do Leste/MT	6	2	0.600 ± 0.129	0.0015 ± 0.0011	4/0
AT	Santa Gertrudes/SP	1	1	1.000 ± 0.000	0.0000 ± 0.0000	0/0
AT	Engenheiro Coelho/SP	1	1	1.000 ± 0.000	0.0000 ± 0.0000	0/0
AT	Ribeirão Preto/SP	12	7	0.909 ± 0.056	0.0013 ± 0.0008	6/1
AT	Campos de Holambra/SP	23	10	0.889 ± 0.037	0.0021 ± 0.0012	14/0
AT	Pedrinhas Paulista/SP	2	2	1.000 ± 0.500	0.0018 ± 0.0021	3/0
AT	Cândido Mota/SP	2	2	1.000 ± 0.500	0.0024 ± 0.0027	4/0
AT	Palmital/SP	1	1	1.000 ± 0.000	0.0000 ± 0.0000	0/0
AT	Alegre/ES	3	1	0.000 ± 0.000	0.0000 ± 0.0000	0/0
AT	Alto Parana/Paraguay	6	4	0.800 ± 0.172	0.0020 ± 0.0014	7/0
AT	Caçador/SC	5	5	1.000 ± 0.127	0.0050 ± 0.0033	18/1
PA	Itaara/RS	3	3	1.000 ± 0.272	0.0077 ± 0.0060	18/1
PA	Cachoeirinha/RS	1	1	1.000 ± 0.000	0.0000 ± 0.0000	0/0
PA	Cachoeira do Sul/RS	1	1	1.000 ± 0.000	0.0000 ± 0.0000	0/0
PA	Pelotas/RS	3	3	1.000 ± 0.272	0.0020 ± 0.0018	5/0
PA	Barra do Ribeiro/RS	6	6	1.000 ± 0.096	0.0047 ± 0.0029	20/1
PA	Osório/RS	2	2	1.000 ± 0.500	0.0030 ± 0.0033	5/0
PA	Camaquã/RS	9	9	1.000 ± 0.052	0.0019 ± 0.0012	12/0
PA	São Lourenço do Sul/RS	2	2	1.000 ± 0.500	0.0091 ± 0.0094	14/1
PA	Monte Redondo/Tucuman/Argentina	2	2	1.000 ± 0.500	0.0030 ± 0.0033	5/0
Puerto Rico	Puerto Rico	4	2	0.667 ± 0.204	0.0016 ± 0.0013	4/0
Total	303	54	0.926 ± 0.006	0.0023 ± 0.0013	49/6

Notes

Nhp = number of haplotypes; Hd = haplotype diversity; *π* = nucleotide diversity; Total groups all localities in one locality.

AM/CE: Amazon Forest transition with Cerrado; AM: Amazon Forest; AT: Atlantic Forest; CE/CA: Cerrado transition with Caatinga; CE: Cerrado; PA: Pampas.

## APPENDIX E

Putative hybrids. Alignment of two distinct sequences of ITS1 obtained from each of the six individuals previously identified by the sequencing of the mtDNA COI gene as *H. armigera* (GenBank accession numbers: MG893729–MG893735 and Appendix F). In the first 10 rows, six sequences of approximately 334 bp are aligned with three ITS1 of *H. zea* and one hybrid obtained in laboratory. In the next eleven lines, six sequences of approximately 147 bp amplified from the same individuals are aligned with ITS1 sequences of *H. armigera* and the same hybrid used above. The shaded area is where the bases are equal, in bold and upper case are the amplicons, hyphen are bases not sequenced or indels and squares with the dashed arrows represent the three primers used. Electrophoresis gel of ITS1 from putative hybrids between *H. armigera* and *H. zea* is shown on the bottom and right. The first sample is one hybrid obtained in laboratory (MG893729 and Appendix F), the next six samples are individuals collected in field that also presented the same two bands pattern relative to *H. armigera* and *H. zea* (confirmed by sequencing: MG893730–MG893735 and Appendix F). The following four samples refer to two *H. zea* and two *H. armigera* individuals which presented the expected band pattern for each species.

## APPENDIX F

Short sequences of six samples that matched >98% with *H. armigera* ITS1: PI10, RR08, RO08, RO28, MS04, RS24, two *H. armigera* from an electrophoresis gel (Appendix E) and one hybrid obtained in laboratory organized in a FASTA file format. Because the sequences are <200 bp, GenBank does not accept their submission. The accession numbers of the sequences that matched >98% with *H. zea* from these samples are MG893729–MG893735.

>*Helicoverpa armigera*/*H. zea* Hybrid born in laboratory, Internal transcribed spacer 1 (ITS1) lower band.

AGGAAGTAAAAGTCGTAACAAGGTTTCCGTAGGGGAACCTGCGGAAGGATCATTAACGTGTAAACGCAAGACGCGACTGGCTCGCGACGCGCGTGTTATAACGTAAACAAAATAATCCACACACCACTAGAGGACACAGAGTCGAAC

>*Helicoverpa armigera*/*H. zea* RO28 Internal transcribed spacer 1 (ITS1) lower band.

CATTAACGTGTAAACGCAAGACGCGACTGGCTCGCGACGCGCGTGTTATAACGTAAACAATAATCC

>*Helicoverpa armigera*/*H. zea* MS04 Internal transcribed spacer 1 (ITS1) lower band.

ATTAACGTGTAAACGCAAGACGCGACTGGCTCGCGACGCGCGTGTTATAACGTAAACAATAATCCACACACCACTAGAGGACACAGAGTCGAACG

>*Helicoverpa armigera*/*H. zea* PI10 Internal transcribed spacer 1 (ITS1) lower band.

ATTAACGTGTAAACGCAAGACGCGACTGGCTCGCGACGCGCGTGTTATAACGTAAACAATAATCCACACACCACTAGAGGACACAGAGTC

>*Helicoverpa armigera*/*H. zea* RR08 Internal transcribed spacer 1 (ITS1) lower band.

TAACGTGTAAACGCAAGACGCGACTGGCTCGCGACGCGCGTGTTATAACGTAAACAATAATCCACACACCACTAGAGGACACAGAGTCG

>*Helicoverpa armigera/H. zea* RS24 Internal transcribed spacer 1 (ITS1) lower band.

CATTAACGTGTAAACGCAAGACGCGACTGGCTCGCGACGCGCGTGTTATAACGTAAACAATAATCCACACACCACT

>*Helicoverpa armigera*/*H. zea* RO08 Internal transcribed spacer 1 (ITS1) lower band.

TAACGTGTAAACGCAAGACGCGACTGGCTCGCGACGCGCGTGTTATAACGTAAACAATAATCCACACACCACTA

>H.armigera_gel_ITS1_Hsp1

AAGTAAAAGTCGTAACAAGGTTTCCGTAGGGGAACCTGCGGAAGGATCATTAACGTGTAAACGCAAGACGCGACTGGCTCGCGACGCGCGTGTTATAACGTAAACAATAATCCACACACCACTAGAGGACACAGAGTCGAACGA

>H.armigera_gel_ITS1_Hs75

TAACGTGTAAACGCAAGACGCGACTGGCTCGCGACGCGCGTGTTATAACGTAAACAATAATCCACACAC
