# Supplementary material for: Invasion origin, rapid population expansion, and the lack of genetic structure of cotton bollworm (Helicoverpa armigera) in the Americas
Source: Ecol Evol. 2019 Jun 17;9(13):7378–401. doi: 10.1002/ece3.5123 (PMC6635935; doi:10.1002/ece3.5123)
Supplement: Supplementary file 1 [file ECE3-9-7378-s001.pdf]

GenBank accession numbers of all samples obtained in this work.

Region	Location	Geographic Coordinates (Long;Lat)	ID	COI - GenBank Accession nº	COII - GenBank Accession nº	Cyt *b* - GenBank Accession nº	Concatenated Haplotypes	ITS1 (Upper band) - GenBank Accession nº	ITS1 (Lower band)**	nDNA-DDC (AL #1, AL #2)	nDNA-RpL11 (AL #1, AL #2)	nDNA-RpS6 (AL #1, AL #2)
CE/CA	Cruz das Almas/BA	-39.101144;-12.667962	BA13	KT828765	KT828994	KT829223	H1	-	-	-	-	-
			BA14	KT828828	KT829057	KT829286	H3	-	-	-	-	-
AT	Santa Gertrudes/SP	-47.532594;-22.457332	SP01	KT828844	KT829073	KT829302	H3	-	-	-	-	-
AT	Engenheiro Coelho/SP	-47.213021;-22.489393	SP02	KT828866	KT829095	KT829324	H4	-	-	-	-	-
AM/CE	Vilhena/RO	-60.149361;-12.750594	RO01	KT828883	KT829112	KT829341	H5	MG893736	-	-	-	-
			RO02	KT828884	KT829113	KT829342	H5	-	-	-	-	-
AT	Ribeirão Preto/SP	-47.810324;-21.170401	SP03	KT828794	KT829023	KT829252	H1	-	-	-	-	-
			SP04	KT828867	KT829096	KT829325	H4	-	-	-	-	-
			SP05	KT828868	KT829097	KT829326	H4	-	-	-	-	-
			SP06	KT828929	KT829158	KT829387	H9	-	-	-	-	-
			SP07	KT828845	KT829074	KT829303	H3	-	-	-	-	-
			SP08	KT828795	KT829024	KT829253	H1	-	-	-	-	-
			SP09	KT828888	KT829117	KT829346	H5	-	-	-	-	-
			SP10	KT828900	KT829129	KT829358	H6	-	-	-	-	-
			SP11	KT828988	KT829217	KT829446	H52	-	-	204, 206	294, 294	265, 268
			SP12	KT828901	KT829130	KT829359	H6	-	-	-	-	-
			SP13	KT828796	KT829025	KT829254	H1	-	-	-	-	-
			SP14	KT828889	KT829118	KT829347	H5	-	-	-	-	-
AT	Campos de Holambra/SP	-48.721898;-23.386681	SP15	KT828846	KT829075	KT829304	H3	-	-	-	-	-
			SP16	KT828911	KT829140	KT829369	H7	-	-	-	-	-
			SP17	KT828797	KT829026	KT829255	H1	-	-	-	-	-
			SP18	KT828869	KT829098	KT829327	H4	-	-	-	-	-
			SP19	KT828798	KT829027	KT829256	H1	-	-	-	-	-
			SP20	KT828989	KT829218	KT829447	H53	-	-	187, 188	294, 294	265, 267
			SP21	KT828821	KT829050	KT829279	H2	-	-	-	-	-
			SP22	KT828847	KT829076	KT829305	H3	-	-	-	-	-
			SP23	KT828822	KT829051	KT829280	H2	-	-	-	-	-
			SP24	KT828823	KT829052	KT829281	H2	-	-	-	-	-
			SP30	MG893742	MG893581	MG893655	H23	-	-	204, 205	294, 294	245, 266
			SP31	KT828826	KT829055	KT829284	H2	-	-	-	-	-
			SP32	KT828920	KT829149	KT829378	H8	-	-	-	-	-
			SP34	KT828849	KT829078	KT829307	H3	-	-	-	-	-
			SP35	KT828990	KT829219	KT829448	H23	-	-	202, 203	294, 294	266, 268
			SP36	KT828991	KT829220	KT829449	H54	-	-	203, 206	293, 294	252, 267
			SP37	KT828912	KT829141	KT829370	H7	-	-	-	-	-
			SP38	KT828850	KT829079	KT829308	H3	-	-	-	-	-
			SP39	MG893743	MG893582	MG893656	H2	-	-	-	-	-
			SP40	MG893744	MG893583	MG893657	H1	-	-	-	-	-
			SP41	MG893745	MG893584	MG893658	H1	-	-	-	-	-
			SP42	MG893746	MG893585	MG893659	H11	-	-	-	-	-
			SP43	MG893747	MG893586	MG893660	H1	-	-	-	-	-
AT	Pedrinhas Paulista/SP	-50.793765;-22.817855	SP25	KT828848	KT829077	KT829306	H3	-	-	-	-	-
			SP26	KT828870	KT829099	KT829328	H4	-	-	-	-	-
AT	Cândido Mota/SP	-50.387800;-22.747570	SP27	KT828953	KT829182	KT829411	H20	-	-	202, 204	294, 294	262, 265
			SP28	KT828824	KT829053	KT829282	H2	-	-	-	-	-
AT	Palmital/SP	-50.218437;-22.786327	SP29	KT828825	KT829054	KT829283	H2	-	-	-	-	-
AM/CE	Itapuã D'Oeste/RO	-63.149000;-9.317500	RO09	KT828981	KT829210	KT829439	H42	-	-	210, 219	294, 294	262, 268
			RO10	KT828982	KT829211	KT829440	H43	-	-	205, 205	294, 294	268, 268
AM/CE	Porto Velho/RO	-63.900430;-8.761160	RO11	KT828885	KT829114	KT829343	H5	-	-	-	-	-
			RO13	KT828865	KT829094	KT829323	H4	-	-	-	-	-
			RO14	KT828983	KT829212	KT829441	H44	-	-	-	-	-
AM/CE	Seringueiras/RO	-63.031406;-11.765456	RO15	KT828790	KT829019	KT829248	H1	-	-	-	-	-
AM/CE	Rolim de Moura/RO	-61.778141;-11.725698	RO16	KT828943	KT829172	KT829401	H11	-	-	-	-	-
			RO24	KT828842	KT829071	KT829300	H3	-	-	-	-	-
AM/CE	Ariquemes/RO	-63.033041;-9.906105	RO17	KT828928	KT829157	KT829386	H9	-	-	-	-	-
AM/CE	Parecis/RO	-61.600612;-12.193709	RO18	KT828840	KT829069	KT829298	H3	-	-	-	-	-
AM/CE	Cerejeiras/RO	-60.817355;-13.187399	RO28	KT828907	KT829136	KT829365	H7	MG893730	Supplementary Material S8.fas	-	-	-
AM/CE	Chupinguaia/RO	-60.902975;-12.555827	RO21	KT828906	KT829135	KT829364	H7	-	-	-	-	-
			RO29	KT828984	KT829213	KT829442	H45	-	-	185, 193	294, 294	265, 268
			RO30	KT828948	KT829177	KT829406	H17	-	-	204, 215	294, 294	269, 269
AM/CE	Espigão D'Oeste/RO	-61.016997;-11.532315	RO22	KT828841	KT829070	KT829299	H3	-	-	-	-	-
			RO23	KT828935	KT829164	KT829393	H10	-	-	-	-	-
			RO31	MG893748	MG893587	MG893661	H9	-	-	-	-	-
AM/CE	Alto Alegre dos Parecis/RO	-61.835502;-12.132361	RO25	KT828955	KT829184	KT829413	H13	-	-	204, 204	294, 294	266, 266
			RO26	KT828957	KT829186	KT829415	H16	-	-	204, 204	294, 294	252, 268
AM/CE	Pimenteiras D'Oeste/RO	-63.580611;-11.505734	RO32	KT828908	KT829137	KT829366	H7	-	-	-	-	-
PA	Itaara/RS	-53.758218;-29.610767	RS01	KT828910	KT829139	KT829368	H7	-	-	188, 206	294, 294	270, 270
			RS02	KT828987	KT829216	KT829445	H48	-	-	204, 209	294, 294	267, 268
			RS03	KT828899	KT829128	KT829357	H6	-	-	204, 212	294, 294	265, 267
CE/CA	Balsas/MA	-45.909556;-7.320222	MA01	KT828893	KT829122	KT829351	H6	-	-	-	-	-
			MA02	KT828853	KT829082	KT829311	H4	-	-	-	-	-
			MA03	KT828945	KT829174	KT829403	H12	-	-	-	-	-
			MA04	KT828830	KT829059	KT829288	H3	-	-	-	-	-
			MA05	KT828959	KT829188	KT829417	H19	-	-	204, 204	294, 294	243, 269
			MA06	KT828770	KT828999	KT829228	H1	-	-	-	-	-
			MA07	KT828831	KT829060	KT829289	H3	-	-	-	-	-
			MA08	KT828832	KT829061	KT829290	H3	-	-	-	-	-
			MA09	KT828833	KT829062	KT829291	H3	-	-	-	-	-
			MA10	KT828771	KT829000	KT829229	H1	-	-	-	-	-
			MA11	KT828834	KT829063	KT829292	H3	-	-	-	-	-
			MA12	KT828930	KT829159	KT829388	H10	-	-	197, 204	294, 294	252, 266
			MA13	KT828874	KT829103	KT829332	H5	-	-	-	-	-
			MA14	KT828772	KT829001	KT829230	H1	-	-	-	-	-
			MA15	KT828952	KT829181	KT829410	H20	-	-	187, 203	294, 294	264, 267
			MA16	KT828913	KT829142	KT829371	H8	-	-	187, 205	294, 294	266, 273
			MA17	KT828773	KT829002	KT829231	H1	-	-	-	-	-
			MA18	KT828774	KT829003	KT829232	H1	-	-	-	-	-
			MA19	KT828950	KT829179	KT829408	H21	-	-	204, 212	294, 294	233, 266
			MA20	KT828775	KT829004	KT829233	H1	-	-	-	-	-
			MA21	KT828960	KT829189	KT829418	H22	-	-	204, 204	294, 294	265, 267
			MA22	KT828938	KT829167	KT829396	H14	-	-	-	-	-
			MA23	KT828776	KT829005	KT829234	H1	-	-	-	-	-
			MA24	KT828968	KT829197	KT829426	H28	-	-	205, 208	294, 294	269, 269
			MA25	KT828854	KT829083	KT829312	H4	-	-	-	-	-
			MA26	KT828807	KT829036	KT829265	H2	-	-	-	-	-
			MA27	KT828875	KT829104	KT829333	H5	-	-	-	-	-
			MA28	KT828956	KT829185	KT829414	H16	-	-	207, 207	294, 294	262, 275
CE	Iraí de Minas/MG	-47.545444;-19.003889	MG01	KT828777	KT829006	KT829235	H1	-	-	-	-	-
AM	Santarém/PA	-54.708170;-2.595665	PA01	KT828926	KT829155	KT829384	H9	MG893737	-	205, 210	294, 294	267, 268
AM	Santarém/PA	-54.676906;-2.521321	PA02	KT828857	KT829086	KT829315	H4	-	-	204, 207	294, 294	266, 269
CE	Monte Carmelo/MG	-47.418806;-18.935333	MG02	KT828914	KT829143	KT829372	H8	-	-	-	-	-
			MG03	KT828808	KT829037	KT829266	H2	-	-	-	-	-
			MG04	KT828961	KT829190	KT829419	H22	-	-	207, 210	293, 294	266, 266
			MG05	MG893749	MG893588	MG893662	H3	-	-	-	-	-
			MG06	KT828894	KT829123	KT829352	H6	-	-	-	-	-
			MG07	KT828947	KT829176	KT829405	H17	-	-	208, 213	294, 294	269, 269
			MG08	KT828895	KT829124	KT829353	H6	-	-	-	-	-
			MG09	KT828809	KT829038	KT829267	H2	-	-	-	-	-
			MG10	KT828925	KT829154	KT829383	H9	-	-	-	-	-
			MG11	KT828778	KT829007	KT829236	H1	-	-	-	-	-
			MG12	KT828915	KT829144	KT829373	H8	-	-	-	-	-
			MG13	KT828810	KT829039	KT829268	H2	-	-	-	-	-
CE	Nova Ponte/MG	-47.687667;-19.048806	MG14	KT828876	KT829105	KT829334	H5	-	-	-	-	-
			MG15	KT828902	KT829131	KT829360	H7	-	-	200, 206	294, 294	265, 267
			MG16	KT828835	KT829064	KT829293	H3	-	-	204, 207	294, 294	266, 266
			MG17	KT828779	KT829008	KT829237	H1	-	-	-	-	-
CE	Costa Rica/MS	-53.129167;-18.543889	MS01	KT828855	KT829084	KT829313	H4	-	-	205, 207	294, 294	265, 267
			MS02	KT828963	KT829192	KT829421	H18	-	-	207, 354	294, 294	265, 267
			MS03	KT828942	KT829171	KT829400	H11	-	-	170, 187	294, 294	268, 270
			MS04	KT828811	KT829040	KT829269	H2	MG893731	Supplementary Material S8.fas	-	-	-
CE/CA	Riachão das Neves/BA	-44.992500;-12.148333	BA15	KT828871	KT829100	KT829329	H5	-	-	-	-	-
			BA16	KT828872	KT829101	KT829330	H5	-	-	-	-	-
			BA17	KT828852	KT829081	KT829310	H4	-	-	-	-	-
			BA18	KT828766	KT828995	KT829224	H1	-	-	-	-	-
			BA19	KT828829	KT829058	KT829287	H3	MG893738	-	-	-	-
CE/CA	Luis Eduardo Magalhães/BA	-45.798333;-12.099444	BA20	KT828944	KT829173	KT829402	H12	-	-	187, 203	269, 294	268, 277
			BA21	KT828924	KT829153	KT829382	H9	-	-	-	-	-
CE/CA	Bom Jesus/PI	-44.359348;-9.071654	PI40	KT828903	KT829132	KT829361	H7	-	-	-	-	-
			PI41	KT828917	KT829146	KT829375	H8	-	-	-	-	-
			PI42	KT828980	KT829209	KT829438	H41	-	-	200, 207	294, 294	246, 269
			PI43	KT828951	KT829180	KT829409	H21	-	-	203, 206	294, 294	269, 269
			PI44	MG893750	MG893589	MG893663	H4	-	-	-	-	-
			PI45	KT828927	KT829156	KT829385	H9	-	-	-	-	-
			PI47	KT828918	KT829147	KT829376	H8	-	-	-	-	-
			PI48	KT828904	KT829133	KT829362	H7	-	-	-	-	-
			PI49	KT828919	KT829148	KT829377	H8	-	-	-	-	-
			PI50	KT828864	KT829093	KT829322	H4	-	-	-	-	-
			PI53	KT828905	KT829134	KT829363	H7	-	-	-	-	-
CE	Paraíso das Águas/MS	-52.968333;-19.052222	MS05	KT828780	KT829009	KT829238	H1	-	-	-	-	-
			MS06	KT828969	KT829198	KT829427	H29	-	-	204, 204	292, 294	262, 268
			MS07	KT828812	KT829041	KT829270	H2	-	-	-	-	-
			MS08	KT828946	KT829175	KT829404	H12	-	-	-	-	-
CE	Rondonópolis/MT	-54.637222;-16.471389	MT08	KT828916	KT829145	KT829374	H8	-	-	-	-	-
			MT09	KT828836	KT829065	KT829294	H3	-	-	-	-	-
			MT10	KT828970	KT829199	KT829428	H30	-	-	204, 205	294, 294	215, 262
			MT11	KT828877	KT829106	KT829335	H5	-	-	204, 208	294, 294	256, 290
			MT12	KT828856	KT829085	KT829314	H4	-	-	-	-	-
CE	Palminópolis/GO	-50.147253;-16.885808	GO01	KT828767	KT828996	KT829225	H1	-	-	-	-	-
CE	Palmeiras de Goiás/GO	-49.968633;-16.873414	GO02	KT828890	KT829119	KT829348	H6	-	-	-	-	-
CE	Palmeiras de Goiás/GO	-50.056753;-16.880353	GO03	KT828768	KT828997	KT829226	H1	-	-	-	-	-
CE	Morrinhos/GO	-49.044558;-17.700333	GO04	KT828891	KT829120	KT829349	H6	-	-	-	-	-
			GO05	KT828962	KT829191	KT829420	H18	-	-	206, 210	294, 294	267, 273
CE	Morrinhos/GO	-48.960928;-17.846294	GO06	KT828937	KT829166	KT829395	H14	-	-	-	-	-
CE	Morrinhos/GO	-48.958908;-17.842750	GO07	KT828769	KT828998	KT829227	H1	-	-	-	-	-
			GO08	MG893751	MG893590	MG893664	H5	-	-	203, 219	294, 294	264, 267
			GO09	KT828804	KT829033	KT829262	H2	-	-	-	-	-
CE	Morrinhos/GO	-48.937964;-17.812383	GO11	KT828805	KT829034	KT829263	H2	-	-	-	-	-
CE	Morrinhos/GO	-48.941742;-17.809753	GO12	KT828958	KT829187	KT829416	H19	-	-	210, 210	294, 294	268, 268
			GO13	KT828892	KT829121	KT829350	H6	-	-	-	-	-
			GO14	KT828954	KT829183	KT829412	H13	-	-	208, 219	294, 294	268, 270
			GO15	KT828806	KT829035	KT829264	H2	-	-	-	-	-
			GO16	KT828873	KT829102	KT829331	H5	-	-	-	-	-
CE/CA	Luis Eduardo Magalhães/BA	-45.871150;-12.397481	BA03	KT828941*	KT829170*	KT829399	H11	-	-	203, 203	294, 294	262, 268
CE/CA	Luis Eduardo Magalhães/BA	-45.868250;-12.559947	BA04	KT828923*	KT829152*	KT829381	H9	-	-	-	-	-
			BA05	KT828802*	KT829031*	KT829260	H2	-	-	-	-	-
CE/CA	Luis Eduardo Magalhães/BA	-45.771319;-12.058325	BA06	KT828965*	KT829194*	KT829423	H25	-	-	-	-	-
CE/CA	Luis Eduardo Magalhães/BA	-45.653700;-12.050444	BA07	KT828966*	KT829195*	KT829424	H26	-	-	-	-	-
CE/CA	Luis Eduardo Magalhães/BA	-45.796317;-12.142786	BA08	KT828763*	KT828992*	KT829221	H1	-	-	-	-	-
CE/CA	Correntina/BA	-44.644361;-13.374594	BA09	KT828851*	KT829080*	KT829309	H4	-	-	-	-	-
CE/CA	Correntina/BA	-44.928069;-13.497653	BA10	KT828764*	KT828993*	KT829222	H1	-	-	-	-	-
			BA11	KT828967*	KT829196*	KT829425	H27	-	-	210, 210	294, 294	262, 266
CE/CA	Correntina/BA	-44.819347;-13.881194	BA12	KT828803*	KT829032*	KT829261	H2	-	-	-	-	-
CE/CA	Rosário/BA	-38.984017;-10.182750	BA01	KT828922*	KT829151*	KT829380	H9	-	-	-	-	-
			BA02	KT828964*	KT829193*	KT829422	H24	-	-	209, 210	294, 294	266, 269
CE/CA	Uruçuí/PI	-44.553992;-7.241091	PI01	KT828878*	KT829107*	KT829336	H5	-	-	-	-	-
			PI02	KT828971*	KT829200*	KT829429	H32	-	-	202, 204	294, 315	262, 268
			PI03	KT828972*	KT829201*	KT829430	H33	-	-	187, 205	294, 294	263, 269
			PI04	KT828934*	KT829163*	KT829392	H10	-	-	-	-	-
			PI05	KT828781*	KT829010*	KT829239	H1	-	-	-	-	-
			PI06	KT828858*	KT829087*	KT829316	H4	-	-	-	-	-
			PI07	KT828896*	KT829125*	KT829354	H6	-	-	-	-	-
			PI08	KT828859*	KT829088*	KT829317	H4	-	-	-	-	-
			PI09	KT828860*	KT829089*	KT829318	H4	MG893739	-	-	-	-
			PI10	KT828816*	KT829045*	KT829274	H2	MG893732	Supplementary Material S8.fas	-	-	-
			PI11	KT828861*	KT829090*	KT829319	H4	-	-	-	-	-
			PI12	KT828973*	KT829202*	KT829431	H34	-	-	205, 205	294, 294	268, 268
			PI13	KT828817*	KT829046*	KT829275	H2	-	-	-	-	-
			PI14	KT828837*	KT829066*	KT829295	H3	-	-	-	-	-
			PI15	KT828782*	KT829011*	KT829240	H1	-	-	-	-	-
			PI16	KT828897*	KT829126*	KT829355	H6	-	-	-	-	-
			PI17	KT828898*	KT829127*	KT829356	H6	-	-	-	-	-
			PI18	KT828783*	KT829012*	KT829241	H1	-	-	-	-	-
			PI19	KT828784*	KT829013*	KT829242	H1	-	-	-	-	-
			PI20	KT828974*	KT829203*	KT829432	H35	-	-	205, 205	294, 294	266, 268
			PI21	KT828975*	KT829204*	KT829433	H36	-	-	203, 212	294, 294	265, 270
			PI22	KT828879*	KT829108*	KT829337	H5	-	-	-	-	-
			PI23	KT828976*	KT829205*	KT829434	H37	-	-	207, 210	294, 294	265, 265
			PI24	KT828785*	KT829014*	KT829243	H1	-	-	-	-	-
			PI25	KT828880*	KT829109*	KT829338	H5	-	-	-	-	-
			PI26	KT828838*	KT829067*	KT829296	H3	-	-	-	-	-
			PI27	KT828881*	KT829110*	KT829339	H5	-	-	-	-	-
			PI28	KT828786*	KT829015*	KT829244	H1	-	-	-	-	-
			PI29	KT828862*	KT829091*	KT829320	H4	-	-	-	-	-
			PI30	KT828818*	KT829047*	KT829276	H2	-	-	-	-	-
			PI31	KT828787*	KT829016*	KT829245	H1	-	-	-	-	-
			PI32	KT828863*	KT829092*	KT829321	H4	-	-	-	-	-
			PI33	KT828882*	KT829111*	KT829340	H5	-	-	-	-	-
			PI34	KT828977*	KT829206*	KT829435	H38	-	-	203, 207	294, 294	266, 266
			PI35	KT828839*	KT829068*	KT829297	H3	-	-	-	-	-
			PI36	KT828788*	KT829017*	KT829246	H1	-	-	-	-	-
			PI37	KT828789*	KT829018*	KT829247	H1	-	-	-	-	-
			PI38	KT828978*	KT829207*	KT829436	H39	-	-	203, 211	294, 294	237, 269
			PI39	KT828979*	KT829208*	KT829437	H40	-	-	207, 207	294, 294	265, 267
AM	Boa Vista/RR	-60.661681;2.895875	RR04	KT828939*	KT829168*	KT829397	H14	-	-	203, 204	294, 294	266, 269
			RR05	KT828986*	KT829215*	KT829444	H47	-	-	204, 206	294, 294	266, 268
			RR06	KT828886*	KT829115*	KT829344	H5	-	-	-	-	-
			RR07	KT828936*	KT829165*	KT829394	H10	-	-	-	-	-
			RR08	KT828887*	KT829116*	KT829345	H5	MG893733	Supplementary Material S8.fas	-	-	-
			RR09	KT828791*	KT829020*	KT829249	H1	-	-	206, 206	292, 294	263, 269
			RR10	KT828949*	KT829178*	KT829407	H17	-	-	206, 210	294, 294	267, 273
			RR11	KT828792*	KT829021*	KT829250	H1	-	-	-	-	-
			RR12	KT828843*	KT829072*	KT829301	H3	-	-	198, 210	294, 294	266, 269

			RR13	KT828909*	KT829138*	KT829367	H7	MG893740	-	-	-	-
			RR14	KT828793*	KT829022*	KT829251	H1	-	-	-	-	-
AM	Bonfim/RR	-59.845772;3.331883	RR01	KT828985*	KT829214*	KT829443	H46	-	-	201, 205	294, 294	262, 268
			RR02	KT828819*	KT829048*	KT829277	H2	-	-	-	-	-
			RR03	KT828820*	KT829049*	KT829278	H2	-	-	-	-	-
PA	Monte Redondo/Tucuman/Argentina	-65.450000;-27.800000	AR01	MG893752	MG893591	MG893665	H2	-	-	204, 206	294, 294	267, 267
			AR02	MG893753	MG893592	MG893666	H15	-	-	202, 222	294, 294	266, 266
CE/CA	Luis Eduardo Magalhães/BA	-45.780538;-12.090454	BA22	MG893754	MG893593	MG893667	H2	-	-	-	-	-
			BA23	MG893755	MG893594	MG893668	H2	-	-	-	-	-
			BA24	MG893756	MG893595	MG893669	H2	-	-	-	-	-
			BA25	MG893757	MG893596	MG893670	H2	-	-	-	-	-
			BA26	MG893758	MG893597	MG893671	H2	-	-	-	-	-
			BA27	MG893759	MG893598	MG893672	H2	-	-	-	-	-
			BA28	MG893760	MG893599	MG893673	H2	-	-	-	-	-
			BA29	MG893761	MG893600	MG893674	H2	-	-	-	-	-
			BA30	MG893762	MG893601	MG893675	H2	-	-	-	-	-
			BA31	MG893763	MG893602	MG893676	H2	-	-	-	-	-
			BA32	MG893764	MG893603	MG893677	H6	-	-	-	-	-
			BA33	MG893765	MG893604	MG893678	H6	-	-	-	-	-
			BA34	MG893766	MG893605	MG893679	H6	-	-	-	-	-
			BA35	MG893767	MG893606	MG893680	H1	-	-	-	-	-
			BA37	MG893768	MG893607	MG893681	H6	-	-	-	-	-
			BA38	MG893769	MG893608	MG893682	H6	-	-	-	-	-
			BA39	MG893770	MG893609	MG893683	H6	-	-	-	-	-
			BA40	MG893771	MG893610	MG893684	H6	-	-	-	-	-
			BA41	MG893772	MG893611	MG893685	H6	-	-	-	-	-
AT	Alegre/ES	-41.532420;-20.762798	ES01	MG893773	MG893612	MG893686	H13	-	-	203, 203	294, 294	268, 269
			ES02	MG893774	MG893613	MG893687	H13	-	-	203, 203	269, 294	269, 269
			ES03	MG893775	MG893614	MG893688	H13	-	-	186, 204	269, 294	268, 269
CE	Santo Antônio de Goiás/GO	-49.309960;-16.481986	GO17	MG893776	MG893615	MG893689	H3	-	-	-	-	-
CE	Primavera do Leste/MT	-54.295055;-15.560519	MT01	KT828813	KT829042	KT829271	H2	-	-	-	-	-
			MT02	KT828931	KT829160	KT829389	H10	-	-	203, 204	294, 294	269, 269
			MT03	KT828932	KT829161	KT829390	H10	-	-	-	-	-
			MT04	KT828814	KT829043	KT829272	H2	-	-	-	-	-
			MT05	KT828933	KT829162	KT829391	H10	-	-	-	-	-
			MT06	KT828815	KT829044	KT829273	H2	-	-	-	-	-
AT	Alto Parana/Paraguay	-54.883533;-24.729080	PG01	MG893777	MG893616	MG893690	H15	-	-	200, 205	294, 294	268, 269
			PG02	MG893778	MG893617	MG893691	H5	-	-	206, 211	294, 294	266, 266
			PG03	MG893779	MG893618	MG893692	H6	-	-	186, 212	294, 294	266, 268
			PG04	MG893780	MG893619	MG893693	H15	-	-	200, 203	294, 294	266, 268
			PG05	MG893781	MG893620	MG893694	H31	-	-	-	-	-
			PG06	MG893782	MG893621	MG893695	H15	-	-	-	-	-
Puerto Rico	Puerto Rico	-66.417950;17.998730	PR01	MG893783	MG893622	MG893696	H16	-	-	206, 206	294, 294	263, 268
			PR02	MG893784	MG893623	MG893697	H16	-	-	206, 206	294, 294	263, 268
Puerto Rico	Puerto Rico	-66.590149;18.220833	PR03	MG893785	MG893624	MG893698	H1	-	-	178, 202	294, 294	268, 269
			PR04	MG893786	MG893625	MG893699	H1	-	-	178, 202	294, 294	268, 269
PA	Cachoeirinha/RS	-51.119972;-29.949111	RS04	MG893787	MG893626	MG893700	H6	-	-	-	-	-
PA	Cachoeira do Sul/RS	-52.944111;-30.224083	RS05	MG893788	MG893627	MG893701	H1	-	-	-	-	-
PA	Pelotas/RS	-52.337589;-31.765399	RS06	MG893789	MG893628	MG893702	H10	-	-	219, 219	294, 294	266, 268
			RS08	MG893790	MG893629	MG893703	H1	-	-	-	-	-
			RS29	MG893791	MG893630	MG893704	H2	-	-	-	-	-
PA	Barra do Ribeiro/RS	-51.304771;-30.300279	RS09	MG893792	MG893631	MG893705	H5	-	-	-	-	-
			RS10	MG893793	MG893632	MG893706	H12	-	-	198, 206	294, 294	263, 269
			RS11	MG893794	MG893633	MG893707	H3	-	-	-	-	-
			RS12	MG893795	MG893634	MG893708	H8	-	-	204, 205	294, 294	266, 269
			RS14	MG893796	MG893635	MG893709	H7	-	-	204, 205	294, 294	245, 266
			RS15	MG893797	MG893636	MG893710	H9	-	-	203, 204	294, 294	246, 269
PA	Osório/RS	-50.269888;-29.888562	RS16	MG893798	MG893637	MG893711	H2	-	-	-	-	-
			RS17	MG893799	MG893638	MG893712	H3	-	-	-	-	-
PA	Camaquã/RS	-51.804831;-30.849398	RS18	MG893800	MG893639	MG893713	H8	-	-	206, 211	292, 294	269, 290
			RS19	MG893801	MG893640	MG893714	H4	-	-	-	-	-
			RS20	MG893802	MG893641	MG893715	H17	-	-	205, 207	294, 294	269, 274
			RS21	MG893803	MG893642	MG893716	H11	-	-	204, 219	294, 294	252, 266
			RS24	MG893804	MG893643	MG893717	H2	MG893734	Supplementary Material S8.fas	-	-	-
			RS25	MG893805	MG893644	MG893718	H14	-	-	205, 209	294, 294	267, 268
			RS26	MG893806	MG893645	MG893719	H3	-	-	-	-	-
			RS27	MG893807	MG893646	MG893720	H1	-	-	-	-	-
			RS28	MG893808	MG893647	MG893721	H50	-	-	204, 206	294, 294	266, 269
PA	São Lourenço do Sul/RS	-51.981800;-31.362850	RS22	MG893809	MG893648	MG893722	H3	-	-	-	-	-
			RS23	MG893810	MG893649	MG893723	H49	-	-	205, 208	294, 294	233, 263
AT	Caçador/SC	-51.012499;-26.776161	SC01	MG893811	MG893650	MG893724	H12	-	-	-	-	-
			SC02	MG893812	MG893651	MG893725	H2	-	-	-	-	-
			SC03	MG893813	MG893652	MG893726	H3	-	-	-	-	-
			SC04	MG893814	MG893653	MG893727	H4	-	-	200, 213	294, 294	266, 266
			SC05	MG893815	MG893654	MG893728	H51	-	-	206, 206	294, 294	266, 266
CE/CA	Palmas/TO	-48.324286;-10.249091	TO01	KT828799	KT829028	KT829257	H1	-	-	196, 205	294, 294	268, 269
			TO02	KT828800	KT829029	KT829258	H1	-	-	-	-	-
			TO03	KT828827	KT829056	KT829285	H2	-	-	206, 222	294, 294	223, 270
			TO04	KT828801	KT829030	KT829259	H1	-	-	-	-	-
			TO05	KT828921	KT829150	KT829379	H8	-	-	203, 203	294, 294	245, 262
			TO06	KT828940	KT829169	KT829398	H14	-	-	206, 206	294, 294	266, 266
-	Luis Eduardo Magalhães/BA	-45.780538;-12.090454	BA36	MG893816	-	-	-	-	-	-	-	-
-	Morrinhos/GO	-48.937964;-17.812383	GO10	MG893817	-	-	-	-	-	-	-	-
-	Paraíso das Águas/MS	-52.968333;-19.052222	MS09	MG893818	-	-	-	-	-	-	-	-
-	Campo Novo Parecis/MT	-57.893046;-13.659014	MT13	MG893819	-	-	-	-	-	-	-	-
-	Sapezal/MT	-58.820371;-13.547942	MT14	MG893820	-	-	-	-	-	205, 215	294, 294	266, 266
-	Marcelândia/MT	-54.521358;-11.084357	MT15	MG893821	-	-	-	-	-	-	-	-
-	Alto Garças/MT	-53.527623;-16.946720	MT16	MG893822	-	-	-	-	-	-	-	-
-	Poxoréu/MT	-54.388760;-15.841449	MT17	MG893823	-	-	-	MG893741	-	-	-	-
-	Primavera do Leste/MT	-54.295055;-15.560519	MT18	MG893824	-	-	-	-	-	-	-	-
-	Bom Jesus/PI	-44.359348;-9.071654	PI46	MG893825	-	-	-	-	-	-	-	-
-			PI51	MG893826	-	-	-	-	-	-	-	-
-			PI52	MG893827	-	-	-	-	-	-	-	-
-			PI54	MG893828	-	-	-	-	-	-	-	-
-	Puerto Rico	-66.590149;18.220833	PR05	MG893829	-	-	-	-	-	-	-	-
-	Vilhena/RO	-60.149361;-12.750594	RO03	MG893830	-	-	-	-	-	-	-	-
-	Vilhena/RO	-60.149361;-12.750594	RO04	MG893831	-	-	-	-	-	-	-	-
-			RO05	MG893832	-	-	-	-	-	-	-	-
-			RO06	MG893833	-	-	-	-	-	-	-	-
-			RO08	MG893834	-	-	-	MG893735	Supplementary Material S8.fas	-	-	-
-	Colorado do Oeste/RO	-60.545945;-13.117843	RO07	MG893835	-	-	-	-	-	-	-	-
-	Porto Velho/RO	-63.900430;-8.761160	RO12	MG893836	-	-	-	-	-	-	-	-
-	Candeias do Jamari/RO	-63.486204;-8.797550	RO19	MG893837	-	-	-	-	-	-	-	-
-	Cerejeiras/RO	-60.817355;-13.187399	RO20	MG893838	-	-	-	-	-	-	-	-
-	Ariquemes/RO	-63.033041;-9.906105	RO27	MG893839	-	-	-	-	-	-	-	-
-	Pelotas/RS	-52.337589;-31.765399	RS07	MG893840	-	-	-	-	-	-	-	-
-	Cachoeirinha/RS	-51.119972;-29.949111	RS13	MG893841	-	-	-	-	-	-	-	-
-	Campos de Holambra/SP	-48.721898;-23.386681	SP33	MG893842	-	-	-	-	-	-	-	-
-	Palmas/TO	-48.324286;-10.249091	TO07	MG893843	-	-	-	-	-	-	-	-
-	-	-	Hybrid born in laboratory	-	-	-	-	MG893729	Supplementary Material S8.fas	-	-	-

*Mastrangelo et al. (2014)
**As the sequences is <200 bp, their submisson is not allowed by GenBank

AM: Amazon Forest; AM/CE: Amazon Forest transition with Cerrado; AT: Atlantic Forest; PA: Pampas; CE/CA: Cerrado transition with Caatinga; CE: Cerrado.
